# Successful Public Speaking Enhances Neural Alignment in Audience Language Networks

**DOI:** 10.1162/NOL.a.218

**Published:** 2026-02-20

**Authors:** Xuanxuan Zhang, Bolong Wang, Linmiao Zhang, Yi Pu, Xiang-Zhen Kong

**Affiliations:** Department of Psychiatry of Sir Run Run Shaw Hospital, Zhejiang University School of Medicine, Hangzhou, China; Department of Psychology and Behavioral Sciences, Zhejiang University, Hangzhou, China; State Key Lab of Brain-Machine Intelligence, Zhejiang University, Hangzhou, China; Shanghai Key Laboratory of Brain Functional Genomics (Ministry of Education), School of Psychology and Cognitive Science, East China Normal University, Shanghai, China

**Keywords:** brain synchronization, functional MRI, intersubject correlation, language network, public speaking

## Abstract

Public speaking is a fundamental form of communication across a wide range of domains; however, the neural mechanisms underlying audience engagement during different speeches remain poorly understood. In particular, it is unclear which functional brain networks support the dynamic fluctuations of audience engagement and what neurobiological processes underlie these effects. In this study, we used naturalistic fMRI combined with intersubject correlation (ISC) analysis to examine how carefully selected and matched speeches, with varying levels of audience engagement, influence neural activity. Our results revealed that the more engaging speech elicited significantly greater interbrain neural synchronization, as indexed by ISC, across a broad range of brain regions. Notably, these engagement-related effects were most prominent in networks associated with language processing and theory of mind, highlighting their critical roles in facilitating shared audience experiences during compelling public communication. A sliding-window analysis further revealed substantial temporal fluctuations in interbrain synchronization throughout the speech. Additionally, neurobiological annotation analyses identified strong associations between engagement-related ISC effects and molecular pathways involved in trans-synaptic signaling, suggesting that intrabrain neuronal communication may contribute to modulating interbrain synchronization. By integrating naturalistic fMRI with ISC analyses, this study offers a promising framework for investigating dynamic neural synchronization among audience members. These findings have broad implications for fields such as education and leadership development, where a deeper understanding of the neural basis of audience engagement could inform strategies to enhance public speaking and communication effectiveness.

## INTRODUCTION

Communication lies at the core of human interaction, enabling the exchange of information, ideas, and emotions between individuals. This process, which connects minds despite physical separation, is governed by shared neurocognitive systems that support mutual understanding (Falk & Scholz, 2018; Hickok, 2014; Mildner, 2008). Public speaking, a widely practiced form of communication spanning domains from education to politics, serves as a powerful tool for informing, inspiring, persuading, and motivating an audience (Lucas, 2004). While some speeches successfully captivate their audience and effectively disseminate knowledge and insights, others struggle to engage their listeners. Understanding the cognitive processes and neurobiological mechanisms underlying audience engagement can provide valuable insights into effective communication as well as education sciences. However, it remains unclear how different speeches affect and engage the brains of audiences, particularly which functional brain networks support the dynamic fluctuations of audience engagement and what neurobiological processes underlie these effects. Understanding these dynamics could offer new insights into how real-world social interactions shape neural responses.

Successful public speaking can be characterized by a speaker’s ability to sustain audience attention, effectively convey ideas, and evoke emotional resonance. Traditional methods for assessing speech effectiveness, such as interviews and self-report questionnaires, primarily focus on post hoc outcomes of the speech experience (Allen et al., 1989; Fotheringham, 1956; Haiman, 1949). While informative, these methods lack objectivity and fail to capture the real-time cognitive and neural responses of the audience, as well as the dynamic nature of audience engagement.

Recent advances in cognitive neuroscience have begun to address these limitations by investigating the neural responses elicited in naturalistic settings. Neuroimaging techniques, such as functional magnetic resonance imaging (fMRI), offer a more objective approach by measuring brain activity in response to real-world stimuli (Nastase et al., 2020; Sonkusare et al., 2019). Research has demonstrated that when individuals process the same information, their neural dynamics can show striking similarities (Hasson et al., 2004, 2010). This phenomenon, referred to as interpersonal neural synchronization, is commonly quantified by intersubject correlation (ISC) analyses of brain activity, reflecting shared perceptual and cognitive processes in naturalistic settings (Hasson et al., 2012; Nastase et al., 2019; Nummenmaa et al., 2018). Public speaking provides a particularly relevant context for this approach, as it represents a one-to-many form of communication in which a single speech is delivered simultaneously to an audience. This raises the question of whether the same speech is processed in a similar way across listeners. ISC offers a direct way to address this question by quantifying the extent to which different brains respond in synchrony to the same speech stream. Prior work has suggested that such neural synchronization can serve as a marker of attention and engagement (Hasson et al., 2012; Nastase et al., 2019; Schmälzle, 2022). By adopting this brain-to-brain perspective, ISC complements traditional GLM- or encoding-based approaches that model responses within individuals, providing a unique lens on how shared engagement unfolds during naturalistic communication.

A growing body of ISC research across diverse contexts, including films, spoken dialogue, narratives, and music, has revealed robust associations between neural synchronization and factors such as physiological arousal (Czepiel et al., 2021), attentional engagement (Cohen et al., 2017; Ki et al., 2016; Song et al., 2021), interest or preference (Dmochowski et al., 2014), emotional responses (Nummenmaa et al., 2012), comprehension (Lahnakoski et al., 2014; Nguyen et al., 2019; Stolk et al., 2016; Yeshurun et al., 2017), and social communication (Li et al., 2024; Schmälzle et al., 2015). For example, studies involving movie viewing and narrative comprehension have reported significant ISC in functionally relevant regions, including the visual and auditory cortices, superior and middle temporal gyri, medial prefrontal cortex, precuneus, and dorsolateral prefrontal cortex (Chang et al., 2021; Hasson et al., 2008; Zacks & Magliano, 2013). These findings suggest that neural synchronization among an audience may serve as a promising marker of audience engagement during public speaking at varying levels of effectiveness.

Audience engagement during public speaking involves a constellation of perceptual and cognitive processes, including visual and auditory sensory processing, linguistic processing, and higher order social cognitive functions (e.g., autobiographical memory and mentalizing). Moreover, different types of public communication, such as persuasive, entertaining, or informative speech, may engage the audience in distinct ways: persuasive and entertaining forms of communication (e.g., political speeches, narratives, and health messages) often rely on rhetorical strategies, storytelling, and emotional appeals to captivate listeners (Imhof et al., 2017, 2020; Ki et al., 2016; Ohad & Yeshurun, 2023; Pérez et al., 2021; Schmälzle et al., 2015); in contrast, informative public speaking, such as educational lectures designed to convey complex, abstract knowledge from expert to novice (Nguyen et al., 2022), demands clarity and precision to facilitate comprehension and retention. Despite the importance and cognitive demands of informative communication, its underlying neural mechanisms remain relatively underexplored.

Furthermore, the dynamic nature of brain activity over the course of a speech has received limited attention. Audience engagement is not static; it fluctuates in response to changes in the speech content, such as the introduction of novel ideas, shifts in narrative style, or emotionally salient moments. Different narrative structures may also have differential cumulative effects over time on the audience. For example, a study examining a suspenseful film demonstrated that the fluctuation of the audience’s brain synchronization tracked their reported levels of suspense (Schmälzle & Grall, 2020). Therefore, understanding the temporal dynamics of neural synchronization, particularly within regions related to language processing and social cognition, can provide deeper insights into how the brain processes and responds to effective communication.

In addition, recent neurogenetic research has suggested potential links between interbrain neural synchronization and neurotransmitter systems. For example, Lotter et al. (2023) applied a multimodal data fusion approach and reported that interpersonal neural synchronization in social contexts is associated with the distributions of GABAergic receptors (Lotter et al., 2023). Based on this work, we reasoned that engagement-related ISC might reflect not only large-scale network dynamics but also the molecular and genetic architecture of the underlying brain regions. Investigating neurobiological processes that mediate the relationship between interbrain neural synchronization and intrabrain neuronal communication will offer a better understanding of how successful public speaking shapes the shared experiences of the audience.

This study adopted a naturalistic fMRI paradigm combined with ISC analysis and aimed to investigate neural synchronization among individuals watching informative speech videos of varying effectiveness. We hypothesized that effective speeches, compared with less effective ones, would evoke higher ISC across a range of brain regions throughout the information-processing hierarchy, from low-level sensory areas to brain regions involved in language processing and social cognition. Next, we employed a sliding-window approach to examine the temporal dynamics of engagement-related neuronal synchronization. We hypothesized that ISC patterns would exhibit dynamic changes as a speech unfolds, with more engaging speeches eliciting longer-lasting and greater ISC. Finally, we performed a series of functional and neurobiological annotation analyses to further interpret our findings, aiming to uncover potential biological processes underlying the observed differences in interbrain neural synchronization.

## MATERIALS AND METHODS

### Participants

A total of 48 young adult participants with normal hearing and normal or corrected-to-normal vision were recruited. Among them, 17 (9 females; age: 21.76 ± 1.75 yr) completed the pre-experiment task for speech video stimuli preparation. This sample size was not pre-specified; rather, it was determined by the number of participants who enrolled in the speech video assessment task (see below). The remaining 31 participants (14 females; age: 22.29 ± 2.84 yr; all right-handed) were recruited to participate the fMRI experiment. Thus, fMRI participants were exposed to each speech only once. Again, this sample size was also determined by participant availability. Previous studies utilizing similar naturalistic stimuli (i.e., videos) and conducting ISC analyses have indicated that such a sample size is sufficient to capture ISCs in neural activity (Imhof et al., 2020; van Baar et al., 2021). All participants were fluent Mandarin speakers and provided written informed consent.

### Speech Video Stimuli Preparation

We adopted a 3-step approach to determine the speech video stimuli used in the fMRI experiment. First, an initial set of 30 speech videos was selected from YiXi Talks (https://yixi.tv/), which is similar to TED Talks (see Table S1 and Table S2 in the Supporting Information, available at https://doi.org/10.1162/NOL.a.218). These speeches are in Mandarin, with similar length (28.64 ± 4.57 min) but covering various topics (e.g., art and design, literature, science). All videos are subtitled. Next, we selected two pairs of candidate speech videos from the initial list based on the following criteria: (1) the two speeches in each pair differ significantly in terms of their level of engagement; (2) the two speeches in each pair share similar topics; (3) the two speech videos in each pair have comparable lengths, with no more than a 2-min difference between them. After that, we recruited a group of participants (*N* = 17, determined by availability) for an independent assessment of these two pairs of candidate speeches. Briefly, participants were instructed to watch these speech videos in sequence and rate their engagement using 18 questions about their engagement (Table S3), covering an overall impression and such specific dimensions as comprehension, agreement of viewpoints, emotional resonance, appearance, facial expression, body language, intonation, pronunciation, speaking rate, persuasiveness, clarity, organization, insight, novelty, vividness, funniness, and colloquialism. Prior research has shown that these factors influence attentional engagement (Chen et al., 2016; Farris et al., 2013; Herbein et al., 2018; Rodero, 2022; Schreiber et al., 2012). To ensure the dimensions were representative and pedagogically meaningful, the final set was selected with guidance from an expert in public speaking education. In this study, we operationalized engagement using the overall impression rating, which was validated against content- and delivery-related dimensions of audience response. Finally, we selected the pair of videos with a larger difference in the overall impression as the stimuli for the subsequent fMRI experiment. The topics of the resulting speeches were related to art and design. We labeled the speech with a higher overall impression score as the Higher Scoring Speech (HSS) and the speech with a lower score as the Lower Scoring Speech (LSS). The lengths of these speeches were nearly identical (24 min 3 s and 23 min 38 s, respectively). These durations were slightly shorter than the original videos available on the official website, as the introductory advertisement segments (10 s for HSS and 50 s for LSS) were removed.

### fMRI Experimental Procedures

In the fMRI experiment, the selected speech videos were presented as stimuli using Psychtoolbox (Kleiner et al., 2007). Videos were projected onto a rear projection screen in the back of the scanner bore. Participants viewed the stimuli through a mirror attached to the head coil. The audio was delivered via MRI-compatible insert earphones. Participants were instructed to keep their heads still and attentively watch the videos. Prior to the scan, they were informed that they would answer a two-option question related to the speech content after each speech video (Table S4). These questions were designed to ensure their attention to the stimuli during the scan. Whole brain fMRI data were recorded while participants watched the speech videos. The order of the two videos was counterbalanced between participants. After scanning, participants were asked to report their experience (e.g., falling asleep and overall feeling of the speeches). One participant was excluded for falling asleep during scanning, and three for incomplete data due to technical issues or late arrival, and five for silent stimuli presentation, leaving 22 participants for the fMRI data analysis. The final sample size is comparable to previous studies using similar naturalistic stimuli and ISC analysis (Imhof et al., 2020; van Baar et al., 2021).

### fMRI Data Acquisition and Preprocessing

Imaging data, including T1-weighted structural and functional scans, were collected using a Siemens MAGNETOM Prisma 3T MRI scanner. High-resolution T1-weighted magnetization prepared rapid gradient echo sequences were first obtained, covering the whole brain with the following parameters: 208 slices, voxel size = 0.90 × 0.90 × 0.90 mm^3^, echo time (TE) = 2.32 ms, repetition time (TR) = 2,300 ms, slice thickness = 0.9 mm, inversion time = 900 ms, flip angle = 8°, and the field of view (FOV) = 240 × 240 mm^2^. Then, during each task session, fMRI images were collected using a gradient echo planar imaging (EPI) sequence with multiband acceleration (TR = 1,000 ms, TE = 34 ms, voxel size = 2.50 × 2.50 × 2.50 mm^3^, voxel matrix = 92 × 92, flip angle = 50°, FOV = 230 × 230 mm^2^, slices number = 52, multiband factor = 4). During the fMRI scanning sessions, participants viewed two videos with durations of 1,443 s and 1,418 s, respectively. Following each video, the scanner remained active for a few seconds while the participants viewed a black screen, resulting in extended scan durations. To ensure consistency across trials, only the first 1,425 s of each scan were included in the analysis.

Preprocessing of fMRI data was carried out using Data Processing and Analysis for Brain Imaging (DPABI; https://rfmri.org/DPABI; Chao-Gan & Yu-Feng, 2010). The preprocessing pipeline included the following steps: (1) conversion of raw DICOM data to NIFTI format, (2) slice-timing correction and head motion correction with rigid body translation and rotation parameters, (3) co-registration of functional images to Montreal Neurological Institute (MNI) space via unified segmentation of the T1-weighted image, (4) removal of linear signal trends over time, (5) regression of nuisance signals, including white matter, cerebrospinal fluid, global signal, and Friston-24 head motion parameters, (6) resampling to an isotropic voxel size of 3 × 3 × 3 mm^3^, (7) application of temporal band-pass filtering (0.01–0.1 Hz), and (8) spatial smoothing using a 6 mm full-width at half-maximum (FWHM) Gaussian kernel. The preprocessed images for each participant were used for the following ISC analysis.

### Intersubject Correlation Analysis and Engagement-Related ISC Differences

For the participant group, we examined interindividual brain synchronization by calculating the ISC for each voxel across the whole brain. The pair-wise ISC for each voxel was defined as Pearson’s correlation between the fMRI time series of a pair of participants. Let *x*_*a*_*i*__ = [*x*_*a*_*i*__(1), …, *x*_*a*_*i*__(*T*)] represent the time series for participant in region *i*, and *x*_*b*_*i*__ = [*x*_*b*_*i*__(1), …, *x*_*b*_*i*__(*T*)] represent the time series for participant *b* in region *i*. The time-averaged ISC was calculated as follows:

$${ISC}_{ab}=\frac{1}{T-1}\sum_{t=1}^{T}\frac{\left(x_{a_{i}}\left(t\right)-\mu_{a_{i}}\right)\left(x_{b_{i}}\left(t\right)-\mu_{b_{i}}\right)}{\sigma_{a_{i}}\sigma_{b_{i}}}$$ (1)

where *μ*_*a*_*i*__ and *μ*_*b*_*i*__ are the means, and *σ*_*a*_*i*__ and *σ*_*b*_*i*__ are the standard deviations of the time series for participant *a* and *b* in region *i*. Note that, unlike resting-state data, these fMRI time series could be aligned between participants based on the onset of each video they watched.

In addition, for each participant, ISC at the individual level was computed by averaging the ISC values between that participant and all other participants. A Fisher’s *r* – *to* – *z* transformation was applied to normalize the correlation values (Abrams et al., 2013). For visualization, individual-level ISC brain maps were averaged across participants to generate group-level ISC maps for both the HSS and LSS conditions. To explore group differences in ISC between the HSS and LSS, a paired *t* test was conducted, with false discovery rate (FDR) correction applied across the brain cortex. Peak *t* values and their corresponding MNI coordinates were taken from the voxel with the maximum *t* statistic in each significant cluster.

### Functional Annotation of the Engagement-Related ISC Differences

We conducted a series of analyses to explore the functional relevance of the engagement-related ISC differences. First, we used the 7-network parcellation from Thomas Yeo et al. (2011) and extracted the averaged ISC values for each network. These networks include the visual, somatomotor, limbic, control, default, dorsal attention, and ventral attention (or salience) networks. Greater ISC difference suggests greater involvement of a given network during more successful public speaking.

Furthermore, we applied the Neurosynth Image Decoder (https://neurosynth.org/decode/; Yarkoni et al., 2011) to decode the cognitive functions associated with the observed ISC differences. Briefly, Neurosynth is a large-scale database of brain activation coordinates derived from published neuroimaging studies. This meta-analytic decoding approach calculates the correlation between the ISC difference map and each of the 1,307 meta-analytic maps in the Neurosynth database, which correspond to various cognitive functions. A higher correlation with a given Neurosynth brain map indicates greater involvement of the associated cognitive function. In addition, we performed the same decoding analysis for the group-averaged ISC maps for both the HSS and LSS conditions.

Given the significant enrichment of language-related functions in the more successful public speaking condition (see Results), we further investigated the language network by conducting a series of analyses focused on lobe-specific effects and the temporal dynamics of ISC.

For the lobe-specific effects, we identified 15 regions within the language network by combining Neurosynth (search term: “language”) with the Schaefer-400 parcellation (>50% cover; Schaefer et al., 2018). Then, we categorized these regions into the frontal and temporal modules and investigated the ISC differences between the HSS and LSS within each module.

To analyze the ISC dynamics within the language network, we utilized a sliding window approach. This method allowed us to examine the temporal fluctuations of ISC over time in each of the 15 regions. A window length L (in TR units) was defined, and ISC was computed within each window. The time-varying ISC at time *t* was calculated as follows:

$${ISC}_{ab}\left(t\right)=\frac{1}{L-1}\sum_{\tau =t}^{t+L-1}\frac{\left(x_{a_{i}}\left(\tau \right)-\mu_{a_{i}}\left(t\right)\right)\left(x_{b_{i}}\left(\tau \right)-\mu_{b_{i}}\left(t\right)\right)}{\sigma_{a_{i}}\left(t\right)\sigma_{b_{i}}\left(t\right)}$$ (2)

where:

$$\mu_{a_{i}}\left(t\right)=\frac{1}{L}\sum_{\tau =t}^{t+L-1}x_{a_{i}}\left(\tau \right)$$ (3)

and

$$\sigma_{a_{i}}\left(t\right)=\sqrt{\frac{1}{L-1}\sum_{\tau =t}^{t+L-1}{\left(x_{a_{i}}\left(\tau \right)-\mu_{a_{i}}\left(t\right)\right)}^{2}}$$ (4)

*μ*_*a*_*i*__(*t*) and *σ*_*a*_*i*__(*t*) are the mean and standard deviation of the time series within the L-length window starting at time *t* in region *i* for participant *a*. Here, we set L = 25 TRs (corresponding to a window duration of 25 s), with a sliding window overlap of five TRs.

To further characterize fluctuations in ISC, we conducted formal statistical testing of the time-resolved ISC series comparing HSS and LSS using cluster-based permutation tests (*n* = 1,000 permutations; *p* < 0.05). This approach controls the family-wise error rate by evaluating temporally contiguous clusters rather than individual time points, consistent with established standards for neuroimaging time-series analysis (Nichols & Holmes, 2002).

In addition, we performed an exploratory annotation of narrative segments corresponding to ISC peaks and troughs. Specifically, we identified high-peak (top 5%) and low-trough (bottom 5%) ISC epochs and manually annotated the associated clips to assess whether fluctuations in ISC aligned with particular narrative events or features of the stimulus. Given the highly similar dynamics of the temporal and frontal regions within the language network (see Figure 5 in the Results), this analysis focused on ISC fluctuations aggregated across the whole network.

### Neurobiological Annotation of the Engagement-Related ISC Differences

The gene expression data were obtained from the Allen Human Brain Atlas (AHBA; Hawrylycz et al., 2012), which is based on microarray technology. This dataset includes brain-wide gene expression and MRI images from six adult donors. Preprocessing of the gene expression data was conducted using the Abagen toolbox (Markello et al., 2021). The preprocessed data were then mapped onto the Schafer-400 cortical atlas (Schaefer et al., 2018). Where gaps in the data existed, expression values were interpolated for regions lacking data by assigning the expression value of the nearest available tissue sample. Due to insufficient microarray samples for the right hemisphere, data from the left hemisphere were mirrored onto the right hemisphere. After preprocessing, we obtained regional gene expression data for a total of 15,633 genes across 400 brain regions.

For each gene, we constructed an expression vector of length 400 corresponding to the 400 cortical regions. Similarly, we extracted a *t* test statistic vector of the same length, representing the ISC differences between the HSS and LSS conditions. We then computed Pearson’s correlation between each gene’s expression vector and the *t* test statistic vector. Genes were ranked in descending order based on their correlation results. Next, we performed a gene set enrichment analysis using GOrilla (https://cbl-gorilla.cs.technion.ac.il/; Eden et al., 2009) on the ranked gene list to identify the neurobiological pathways potentially involved in the HSS-induced differences in interindividual brain synchronization. Briefly, gene set enrichment analysis evaluates whether predefined sets of genes, such as those associated with particular biological functions or signaling pathways, are statistically overrepresented among the top-ranked genes. This approach allows us to move beyond single-gene associations and to highlight broader molecular mechanisms that may underlie engagement-related ISC effects. We included gene ontology (GO) categories for biological process, molecular function, and cellular component. The statistical significance was set at *p*_FDR_ < 0.05 for multiple comparison correction. By linking ISC with gene expression maps, we aimed to provide converging evidence that molecular substrates involved in inhibitory–excitatory balance are relevant for audience engagement.

In addition, we conducted similar analyses with neurotransmitter systems. We used neuromaps (Version 0.0.5; https://github.com/netneurolab/neuromaps; Markello et al., 2022) and extracted brain maps of 19 neurotransmitter receptor/transporters from publicly available PET images. These maps included Serotonin receptor subtypes such as 5-HT_1A_ and 5-HT_4_; NMDA: Nmethyl-D-aspartate receptor; D1: dopamine receptor; D2: dopamine receptor; mGluR5: metabotropic glutamate receptor 5; DAT: dopamine transporter; M1: muscarinic acetylcholine receptor; CB1: Cannabinoid receptor 1; *α*4*β*2: alpha-4 beta-2 nicotinic receptor; NET: norepinephrine transporter; mu: mu-opioid receptor; VAChT: Vesicular acetylcholine transporter; H3: histamine receptor; 5-HTT: serotonin transporter. We then computed spatial Pearson’s correlations between each neurotransmitter map and the ISC map separately for the HSS and LSS conditions. To test whether these correlations differed significantly between conditions, we applied Fisher’s *Z* test (*p* < 0.05).

## RESULTS

**Figure 1.**
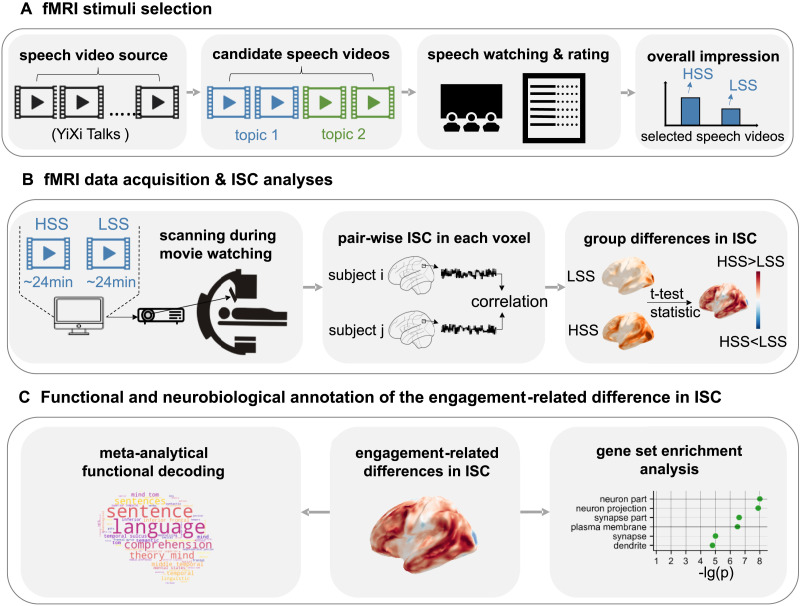
Study procedure and ISC analysis. (A) Schematic of fMRI stimuli preparation. A multiple-step approach was employed to determine the two speech videos with differential engagement levels selected from a larger set of candidates. YiXi Talks is a Chinese platform similar to TED. (B) Schematic of fMRI data acquisition and ISC analyses. fMRI data were collected when participants were watching the obtained speech videos. We calculated the ISC maps for the HSS and LSS separately. Then, a *t* test was employed to compare the ISC maps between the two conditions. (C) Neurobiological and functional annotation of the engagement-related differences in ISC. We applied the Neurosynth Image Decoder to decode the cognitive functions associated with the ISC differences. To further identify biological processes underlying engagement-related changes in ISC, we employed whole-brain spatial association analyses with gene expression patterns. HSS: higher scoring speech, LSS: lower scoring speech, ISC: intersubject correlation.

We employed a 3-step procedure to carefully select and match speech videos for fMRI data collection (Figure 1A). Briefly, first we compiled an initial set of 30 speech videos from YiXi Talks, a high-quality speech platform analogous to TED Talks (Table S1). Next, two authors (X.Z. and L.Z.) selected two pairs of candidate videos, each matched in length and topic, for further assessment (Table S2). Finally, we conducted a detailed evaluation with a group of participants (*N* = 17) and selected the final two speeches as stimuli for the fMRI experiment. For further details on this procedure, please refer to Materials and Methods.

**Figure 2.**
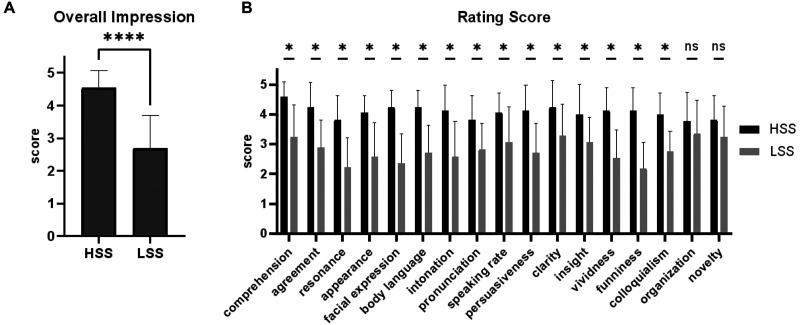
Rating results of the speech videos. (A) Overall impression ratings for HSS and LSS. (B) Ratings of HSS and LSS on the remaining 17 sub-items of the evaluation questionnaire. The results showed significant differences in scores between these two speeches on most sub-items (paired *t* test, *: *p* < 0.05).

This screening procedure resulted in a pair of speech videos that differed significantly in overall impression ratings (Figure 2A; *t* = 6.080, *p* < 0.0001, Cohen’s *d* = 1.473) as well as in scores for most questionnaire items (Figure 2B). These videos were termed Higher Scoring Speech (HSS) and Lower Scoring Speech (LSS) and were used in the subsequent fMRI data collection.

### ISC of Brain Activity When Participants Watched Public Speech Videos

**Figure 3.**
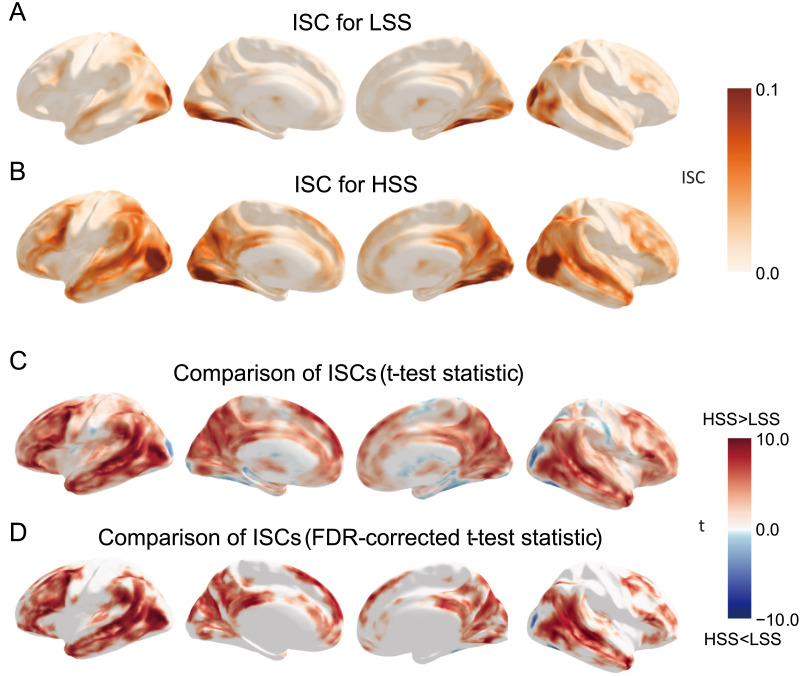
ISC maps and statistical comparison results. (A) ISC map for the LSS condition. (B) ISC map for the HSS condition. (C) Unthresholded *t*-test statistical map comparing ISC between HSS and LSS. (D) The *t*-test statistical map after FDR correction.

We collected fMRI images when participants watched the HSS and LSS videos, with the order of presentation counterbalanced between participants. The ISC analysis showed relatively high ISC in the primary visual cortex and ventral temporal regions for both the LSS (Figure 3A) and HSS (Figure 3B) conditions, indicating synchronized neural responses in the sensory systems across individuals. These results confirm that participants followed experimental instructions and remained attentive to the video stimuli during the fMRI sessions. Beyond these sensory regions, the HSS condition elicited additional high ISC in multiple higher order regions, including the inferior frontal gyrus (IFG), middle temporal gyrus (MTG), and cuneus (Figure 3B).

### Engagement-Related Differences in ISC

Next, we compared quantitatively ISC maps between the HSS and LSS conditions using a paired *t* test. Our results showed that the HSS led to significantly higher ISC across various cortical regions, including the IFG (peak voxel *t* = 5.20, *p* = 1.60e−08; MNI coordinate: 54, 30, −3), MTG (peak voxel *t* = 10.23, *p* = 6.50e−10, MNI coordinate: −42, −66, 6), and cuneus (peak voxel *t* = 8.48, *p* = 3.20e−8; MNI coordinate: 18, −63, 25; Figure 3D; FDR-corrected *p* < 0.05). These regions overlapped considerably with both the language network and the posterior part of the default mode network (DMN), suggesting increased engagement of language processing and DMN-related functions during the viewing of HSS, which left a stronger impression.

In addition, we identified small clusters within the visual system that exhibited significantly higher ISC for the LSS compared to the HSS, such as the left (peak voxel *t* = −6.61, *p* = 1.52e−06; MNI coordinate: −36, −66, −15) and right (peak voxel *t* = −8.64, *p* = 2.35e−08; MNI coordinate: 33, −41, −21) fusiform gyri and occipital areas (peak voxel *t* = −6.34, *p* = 2.75e−06; MNI coordinate: 33, −81, 3), all of which are essential for visual object recognition (FDR-corrected *p* < 0.05). These results suggest that audiences might prioritize visual information (e.g., speaker’s face and other visual cues) when watching a speech that leaves a relatively weaker impression.

### Functional Annotation of the Engagement-Related ISC Differences

We conducted a series of analyses to explore the functional relevance of the engagement-related ISC differences.

#### The network-level analysis

**Figure 4.**
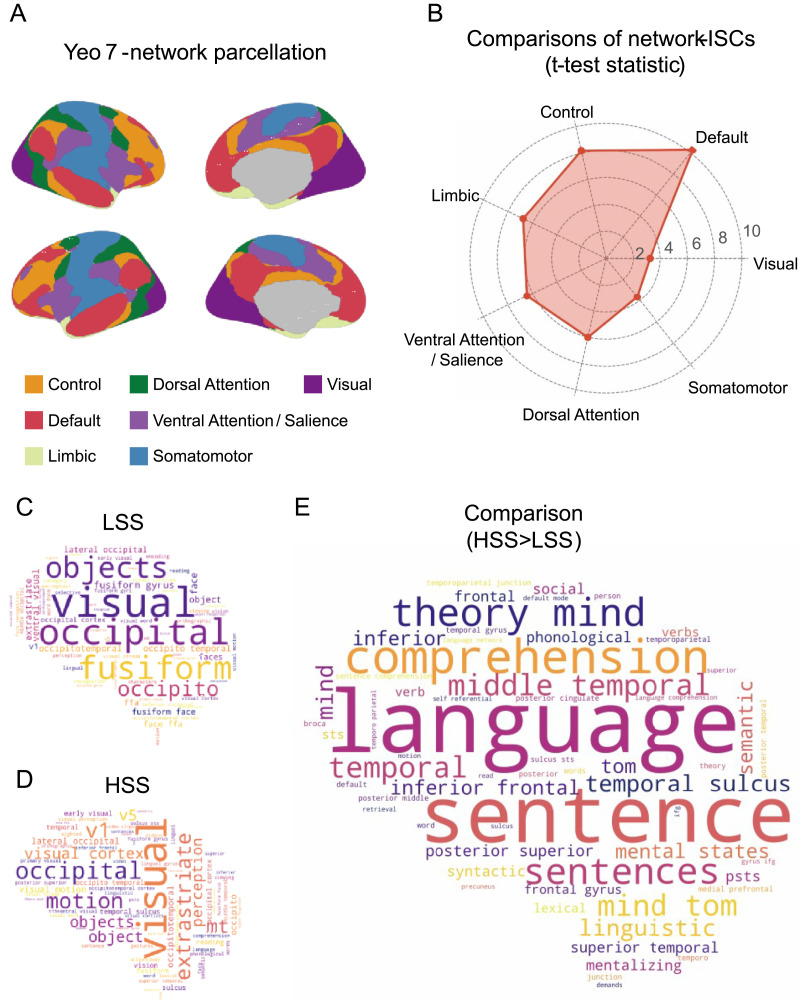
Functional annotation of the engagement-related ISC differences. (A) The network parcellation used for the network-level analysis (Thomas Yeo et al., 2011). (B) Network-level comparisons of the averaged ISC between the HSS and LSS conditions. (C) Functional decoding results of the ISC map for the LSS condition. (D) Functional decoding results of the ISC map for the HSS condition. (E) Functional decoding results of the engagement-related ISC differences (HSS > LSS).

Using the 7-network parcellation of the cortex (Figure 4A), we found the largest ISC differences in the DMN (*t* = 10.29, *p* = 1.16e−09; Cohen’s *d* = 1.81) and the control network (*t* = 8.18, *p* = 5.73e−08; Cohen’s *d* = 1.74; Figure 4B). Meanwhile, the visual network (*t* = 3.24, *p* = 0.004; Cohen’s *d* = 0.65) and the somatomotor network (*t* = 3.68, *p* = 0.001; Cohen’s *d* = 0.79) exhibited the smallest engagement-related differences in ISC (Figure 4B).

#### Neurosynth-based functional decoding

We applied the Neurosynth Image Decoder to characterize the relevant functions underlying the ISC patterns across the brain for both HSS and LSS conditions, as well as the engagement-related differences between the two (see Materials and Methods). As expected, the ISC maps for both HSS and LSS showed strong correlations with terms related to the visual system, such as “visual” and “occipital” (Figure 4C–D). More importantly, we found that the *t* test statistic map, indicating the engagement-related differences in ISC, showed the highest correlations with terms related to language processing and theory of mind (e.g., “language”: *r* = 0.255, “comprehension”: *r* = 0.241, and “theory of mind”: *r* = 0.238). These results underscore the considerable involvement of language comprehension and theory of mind in the increased ISC observed in the HSS condition. Interestingly, the *t*-test statistic map also displayed pronounced negative correlations with terms such as “fusiform gyrus” (*r* = 0.191) and “face” (*r* = −0.165), though the strength of these correlations was weaker than those positive correlations related to language functions (Table S5). Nonetheless, these findings further support our earlier speculation regarding the role of face processing in the increased ISC observed in the LSS condition.

#### Temporal dynamics of ISC within the language network

**Figure 5.**
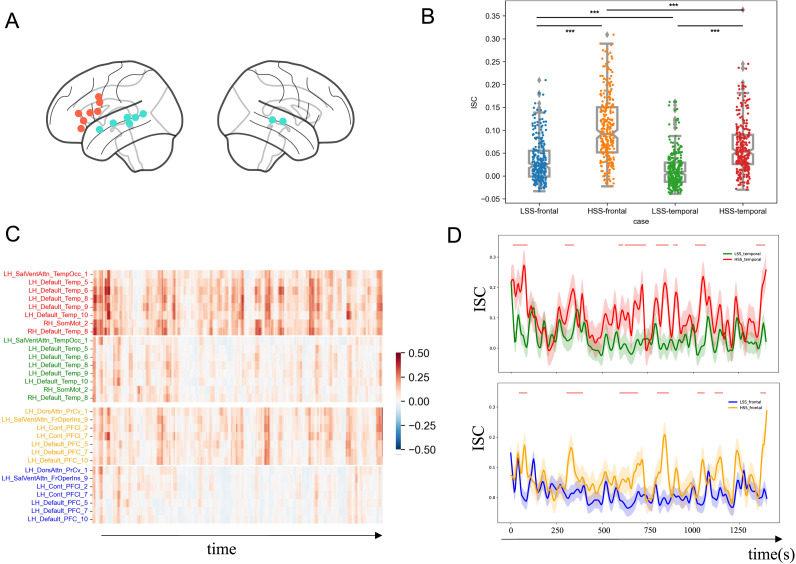
Temporal dynamics of ISC within the language network. (A) Spatial distribution of the 15 ROIs within the language network. Red spots indicate ROIs located in the frontal lobe, while blue spots denote ROIs in the temporal lobe. (B) Comparisons of ISC across different conditions (i.e., HSS vs. LSS) and region sets (i.e., frontal vs. temporal). Each spot represents participants’ averaged ISC across the ROIs in the temporal or frontal sets within a time window. (C) The dynamics of sliding-window ISC in the 15 ROIs exhibited fluctuations over time for both conditions. The top half of the panel illustrates ISC dynamics within the temporal lobe for the HSS (red) and LSS (green), while the bottom half shows the ISC dynamics within the frontal lobe for the HSS (yellow) and LSS (blue). (D) Averaged ISC within the temporal and frontal regions of the language network throughout the speeches. The shaded regions depict the 95% confidence intervals. The red bars annotate the time windows that differentiate two speeches (using a cluster-based permutation test, cluster level *p* < 0.05).

Given the significant involvement of language-related functions in the more successful public speech, we conducted additional analyses focusing on regions within the language network. Specifically, we identified 15 regions of interest (ROIs) within the language network based on functional activation during language-related tasks (Figure 5A; see Materials and Methods) and grouped them into two sets: frontal and temporal regions. ISC was calculated using a sliding-window approach for each ROI and then averaged separately for the frontal and temporal sets. As expected, both sets showed significantly higher ISC in the HSS condition compared to the LSS condition (frontal: *t*(281) = 14.29, *p* = 9.98e−40; temporal: *t*(281) = 12.08, *p* = 5.29e−30). More importantly, when comparing ISC between the temporal and frontal sets, significant differences were observed for both HSS (temporal > frontal: *t*(281) = 7.56, *p* = 1.63e−13) and LSS (temporal > frontal: *t*(281) = 5.51, *p* = 5.58e−08) (Figure 5B). These findings indicate that although both temporal and frontal regions contribute critically to comprehension during public speaking, the temporal lobe may play a more prominent role in these processes.

**Figure 6.**
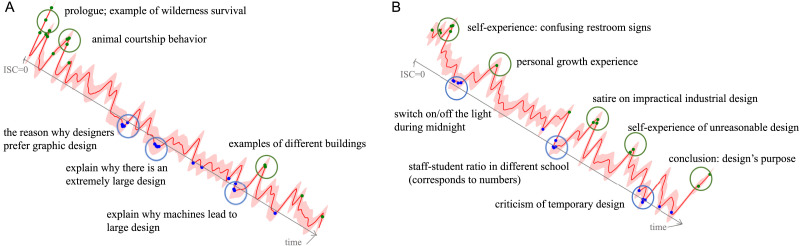
Semantic annotation results for the HSS (A) and LSS (B) conditions in the language network. The shaded regions depict the 95% confidence intervals. Colored peaks and toughs indicate the top and bottom time points during the speech, respectively.

Focusing on the temporal changes in ISC, we observed several notable patterns. First, all 15 ROIs exhibited significant fluctuations in ISC over time for both HSS and LSS conditions (Figure 5C; for ISC dynamics across the whole brain, see Supplementary Video 1). Additionally, fluctuation patterns were temporally aligned between the temporal and frontal regions (Figure 5D), suggesting sustained temporal synchrony of engagement-related ISC dynamics within the language network at the timescale of our analysis. Cluster-based permutation testing revealed multiple clusters of significantly stronger ISC in the HSS compared with the LSS condition (Figure 5D). While no substantial differences were observed at the very beginning of the speeches, significant differences emerged around 10 s in temporal regions and 60 s in the frontal regions, indicating that engagement-related ISC differences develop rapidly (Figure 5D). Finally, exploratory annotation of ISC peaks and troughs showed that high ISC epochs coincided with novel examples and engaging stories, whereas low ISC epochs corresponded to abstract theories or less captivating contents, a consistent pattern across both HSS and LSS conditions (Figure 6 and Table S6). These findings suggest that narrative features shape neural synchrony (see Discussion).

### Neurobiological Annotation of the Engagement-Related ISC Differences

**Figure 7.**
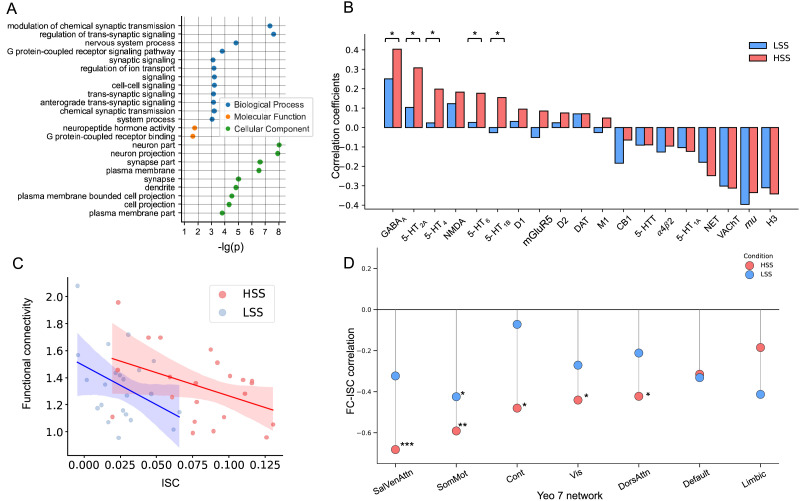
Neurobiological annotation of the engagement-related ISC differences. (A) Gene expression associations. The results of gene set enrichment analysis are arranged by different gene ontology (GO) terms. Blue spots indicate GO terms of biological process, orange spots indicate GO terms of molecular function, and green spots indicate GO terms of cellular component. The −log(p) values represent statistical significance of the enrichment, with higher values indicating stronger evidence that a given gene set is associated the observed ISC pattern. (B) Spatial correlation coefficients between neurotransmitter maps and ISC maps. A Fisher’s *Z* test was performed to assess whether the correlation coefficients differed significantly between the HSS and LSS conditions (*p < 0.05). (C) Correlation between functional connectivity (FC) and ISC within the language network. Each spot indicates an individual’s averaged FC strength and averaged ISC across the 15 ROIs in the language network. Red spots denote the HSS condition, and blue spots denote the LSS condition. The shaded regions depict the 95% confidence intervals. (D) Correlation between FC and ISC for each of the Yeo-7 networks. SalVentAttn = salience/ventral attention; SomMot = somatomotor; Cont = frontoparietal control; Vis = visual; DorsAttn = dorsal attention; Default = default mode; Lim = limbic. (*p < 0.05; **p < 0.005; ***p < 0.001).

Finally, we performed a series of additional analyses to identify potential biological processes underlying the engagement-related ISC differences. Our whole-brain spatial association analyses with gene expression patterns revealed differential correlations with specific genes (e.g., CAPN12: *r* = 0.427, *p* = 3.67e−19; NECTIN1: *r* = 0.369, *p* = 2.47e−14). Gene set enrichment analysis of the ranked genes showed significant associations between the engagement-related changes in ISC and various GO terms (FDR-corrected *p* < 0.05; Figure 7A). For example, the top GO terms related to biological process included “modulation of chemical synaptic transmission” (enrichment = 2.57, FDR-corrected *p* = 4.16e−8) and “regulation of trans-synaptic signaling” (enrichment = 2.56, FDR-corrected *p* = 2.31e−8); and the most enriched GO terms related to cellular component included “neuron part” (enrichment = 1.77, FDR-corrected *p* = 9.34e−9) and “neuron projection” (enrichment = 2.05, FDR-corrected *p* = 1.42e−8).

In addition, a recent study reported a positive association between interpersonal neural synchronization and neurotransmitter systems, particularly the GABAergic (GABA_A_) receptor (Lotter et al., 2023). Consistent with this finding, our results revealed a positive correlation between ISC maps and the distribution of GABA_A_ receptors in both the HSS (*r* = 0.40) and LSS (*r* = 0.25) conditions (Figure 7B). More importantly, a Fisher’s *Z* test showed that the GABA_A_-ISC association was significantly stronger in the HSS condition than in the LSS condition (*p* = 0.03), suggesting GABA-regulated signal transmission as a potential biological mechanism underlying the engagement-related differences in ISC. In addition, we found similar patterns for the serotonin receptors 5-HT_6_, 5-HT_4_, 5-HT_2A_, 5-HT_1B_, and 5-HT_1A_ (Figure 7B). These neurotransmitter receptors are primarily involved in serotonergic signaling and are crucial for regulating various physiological and psychological functions (e.g., learning and memory, and emotion regulation). Taken together, these results indicate that within-brain interneuronal communication may underlie the engagement-related changes in ISC observed in our fMRI study.

Finally, as an initial step toward testing the hypothesis that intrabrain neural communication (functional integration) is linked to interbrain neural synchronization, we investigated the relationship between FC and ISC within the language network. Specifically, we examined whether individual differences in FC within the language network were associated with variations in ISC. Our results revealed a trend toward lower ISC among participants with greater FC in the language network (*Z* = −2.76, *p* = 0.006). This negative association was observed in both the HSS (*r* = −0.435, *p* = 0.04) and LSS (*r* = −0.406, *p* = 0.06) conditions (Figure 7C). These findings suggest that greater functional integration within an individual’s language network may reduce the extent to which their brain synchronizes with others.

To examine whether the negative correlation between ISC and FC was specific to the language network, we extend our analysis to all seven canonical Yeo networks. Consistent with the language-network results, negative ISC–FC correlations were also observed across these networks, particularly for the HSS condition (Figure 7D). The strongest correlations emerged in the salience/ventral attention network (SalVentAttn; HSS: *r* = −0.682, *p* = 0.000473), the somatomotor network (SomMot; HSS: *r* = −0.591, *p* = 0.00376), and the frontoparietal control network (Cont; HSS: *r* = −0.480, *p* = 0.0237). Taken together, these findings provide further evidence for a relationship between intrabrain functional integration and interbrain neural synchronization.

## DISCUSSION

Understanding the psychological and neurobiological mechanisms underlying audience engagement during informative public speaking provides valuable insights into effective communication and education practices. The present study investigated the neurobiological correlates of successful public speaking by examining interbrain synchronization (measured by ISC) as participants watched two speeches varying in levels of engagement. Our results revealed that the HSS, compared to the LSS, elicited greater, more widespread, and more sustained ISC, particularly in brain regions associated with language processing and theory of mind. Furthermore, neurobiological annotation analyses point to intrabrain neural communication (e.g., molecular pathways related to trans-synaptic signaling) as a potential factor underlying interbrain synchronization, a possibility that warrants further investigation. These findings provide new insights into how public speaking can synchronize brain activity across individuals, establishing a foundation for future research into the neural underpinnings of social influence and cohesion, particularly in real-world communicative contexts.

### Effective Speeches Evoke Neural Synchronization, Especially in Language and Social Cognition Networks

Our ISC analysis revealed that both speeches evoked considerable interbrain neural coupling across a wide range of brain regions. This coupling spanned the information-processing hierarchy, from low-level sensory regions to higher order language and social cognition areas. Compared to the LSS, the HSS generated greater ISC in regions associated with linguistic processing and social cognition. Specifically, brain regions such as the MTG, IFG, and angular gyrus, which are crucial for processing linguistic information and understanding others’ intentions, beliefs, and emotions (Davey et al., 2016; Noonan et al., 2013; Seghier, 2013), showed heightened ISC. These results align with the hypothesis that effective public speaking engages both language processing regions and social cognitive networks, suggesting that effective speeches are more likely to synchronize neural activity in regions involved in interpreting verbal content and inferring others’ mental states.

Additionally, the pronounced engagement of central hubs of the DMN, including the precuneus, angular gyrus, temporoparietal junction, and posterior cingulate cortex (PCC), during the HSS implicates a compelling link between effective speaking and self-referential processing (Raichle, 2015). These results are consistent with previous studies showing that narrative speech comprehension involves an extended network, including the DMN and bilateral IFG (Grall et al., 2021; Wilson et al., 2008). Our findings also extend those of Schmälzle et al. who found that rhetorically powerful political speeches generate stronger neural alignment in language-processing regions, such as bilateral superior temporal gyri and medial prefrontal cortex (Schmälzle et al., 2015). In contrast to prior research centered on static language processing, we emphasize the importance of dynamic neural activity during live speech delivery, where audience engagement is modulated by a combination of verbal and nonverbal signals.

Interestingly, our findings diverge slightly from previous research on the persuasive effectiveness of health messages (Imhof et al., 2017, 2020), which showed that more effective video messages evoked heightened ISC in the insula, a region associated with processing of emotionally salient information and risk perception. Effective videos also engaged sensory cortices, PCC, precuneus, and dorsomedial prefrontal cortex, consistent with our findings. These results emphasize the distinction between persuasive and informative speeches, with the former focusing more on emotional impact and the latter more on effective information delivery, similar to instructional settings. For example, Nguyen et al. (2022) observed teacher–student neural coupling in sensory and language regions, including the superior temporal sulcus and higher level areas such as the posterior medial cortex and superior parietal lobule, during educational lectures.

### Effective Speeches Evoke Sustained Neural Synchronization

Moreover, interbrain synchronization may fluctuate in response to speeches with distinct narrative structures. A computational analysis of narrative trajectories in public speaking showed that variations in the emotional, linguistic, and social elements are key to audience engagement, rather than a flat delivery (Zeng et al., 2023). In terms of neural synchronization dynamics, a study using a suspenseful film revealed that audience brain alignment fluctuated over time, tracking self-reported levels of suspense, especially in regions associated with salience processing and executive control (Schmälzle & Grall, 2020). Given the importance of language comprehension during speech watching and the brain’s dynamic nature, we further explored the temporal variation of ISC within the language network using sliding-window ISC analysis. Our results exhibited time-varying fluctuations in neural synchronization for both speeches, highlighting the evolving nature of audience engagement.

Our time-resolved ISC analyses further highlight the dynamic nature of audience engagement during public speaking. We found that ISC differences between the high- and low-engagement speeches (i.e., HSS vs. LSS) emerged early, several seconds after the onset, and fluctuated over time (Figure 5). This temporal profile aligns with the literature on thin-slice judgments and the dynamics of audience attention, which emphasizes that audiences form rapid impressions of a speaker’s effectiveness within brief initial exposures (Schmälzle et al., 2025; Todorov, 2017). Although exploratory, these findings suggest that sliding-window ISC can serve as a neural index of how engagement unfolds over time, thereby linking the dynamics of interbrain coupling with established theories of impression formation and audience attention in public speaking.

Notably, the HSS elicited significantly higher ISC than the LSS at multiple time segments throughout the speech (see Figure 5C–D, cluster level *p* < 0.05). Periods of heightened ISC often coincided with the delivery of novel examples, engaging stories, or topic transitions (Schmälzle et al., 2022, 2024). Similarly, previous research using dynamic intersubject functional correlation (ISFC) analysis found that the homogeneity in ISFC state expression in typically developing participants was negatively correlated with the contextual shift from “fun” to “science” scenes in a movie, indicating more aligned brain responses during the “fun” scenes (Bolton et al., 2020). Taken together, these findings suggest that organizing information within a coherent narrative structure, through the use of socially engaging examples, story elements, and meaningful transitions, can promote shared neural processing across listeners and enhance collective engagement (Martinez-Conde et al., 2019; Willems et al., 2020), as also supported in prior research on naturalistic communications (Schmälzle et al., 2022, 2024).

Overall, temporal fluctuations in brain alignment across the audience were linked to specific speech or movie cues. Moments of idiosyncratic neural responses may reflect individual differences in cognitive strategies or attentional focus, while periods of heightened synchronization—corresponding to engaging content—may recruit broader cognitive and attentional resources, facilitating deeper cognitive and social engagement. These varying levels of engagement indicate that a speaker’s ability to effectively deliver viewpoints and integrate tangible examples or stories is crucial in enhancing neural synchronization among the audience and, therefore, the overall effectiveness of the speech.

### Neurobiological Correlates of Engagement-Related Neural Synchronization

A recent study reported a positive association between interpersonal neural synchronization and neurotransmitter systems, particularly the GABAergic (GABA_A_) receptor (Lotter et al., 2023). This provides a testable hypothesis regarding its neurobiological underpinnings of the observed engagement-related ISC effects. Notably, GABAergic neurotransmission has been implicated in gain control, excitatory/inhibitory balance, dynamic range modulation of cortical circuits, and the synchronization and generation of cortical rhythms (or oscillations). It is also associated with numerous cognitive functions, including attention, memory, cognitive flexibility, and social cognition (Chattopadhyaya & Di Cristo, 2012; Ferguson & Gao, 2018; Schmidt-Wilcke et al., 2018).

Through further neurobiological annotation analyses, we identified critical biological pathways, such as modulation of chemical synaptic transmission and regulation of trans-synaptic signaling, that underlie the engagement-related ISC differences. Importantly, we found a positive correlation between ISC maps and the distribution of GABA_A_ receptors in both the HSS and LSS conditions. Our results showed that this GABA_A_-ISC association was significantly stronger in the HSS condition than in the LSS condition, suggesting that GABA-regulated signal transmission may be a potential biological mechanism driving engagement-related changes in ISC. These results imply that alterations in within-brain interneuronal communication could play a critical role in the observed ISC differences.

As a preliminary step toward testing this hypothesis, we investigated the correlation between FC and ISC within the language network. Our analysis revealed significant negative correlations in both speech videos, indicating that individuals with lower FC within the language network exhibited higher synchronization with others. This finding aligns with the idea that cognitive systems in the brain become more integrated and less segregated to facilitate information transfer across distributed functional networks (Achard & Bullmore, 2007; Fox et al., 2005). In this context, decreased functional integration within the language network may promote the coordination of whole-brain activity, enabling the efficient processing of rich and dynamic information in public speaking, thereby contributing to elevated neural synchronization among the audience. Overall, our findings suggest that flexible interactions between distributed brain regions may underpin the construction of coherent mental representations during naturalistic cognition.

While these neurobiological annotation analyses are necessarily preliminary and hypothesis-generating, they highlight candidate pathways—such as GABAergic systems and synaptic transmission—that could guide future work at the intersection of social neuroscience, molecular mechanisms, and communication research.

### Implications and Limitations

This study is part of a broader investigation into the neurobiological mechanisms underlying audience engagement during informative speeches. To our knowledge, it is the first to examine this process from the audience’s perspective using naturalistic neuroimaging. This approach offers new insights into the neural correlates of successful public speaking. For example, we found that significant differences in interpersonal neural synchronization (indexed by ISC) emerged as early as one minute after the speech began, suggesting that engagement-related neural signatures appear rapidly.

Our preliminary annotation of narrative segments suggests that novel examples and engaging stories coincided with higher ISC. While these findings resonate with prior work on effective communication, we do not make direct recommendations for instructional practice. Instead, our primary contribution is to demonstrate that ISC provides a neural measure of audience engagement during naturalistic public speaking. Establishing ISC as a sensitive index of engagement carries practical implications in that it can serve as an objective tool to complement existing behavioral and self-report approaches for evaluating and potentially improving public speaking and teaching practices. We recognize that more systematic analyses will be required before drawing feature-specific recommendations.

While this study offers valuable insights, several limitations should be acknowledged. The restricted number of speech stimuli—one with a relatively higher engagement score and one with a lower engagement score—may limit the generalizability of the findings. This constraint arises from potential differences in variables beyond audience engagement, such as speaker volume and video quality. Although we carefully selected and matched the stimuli to minimize these confounding factors, residual differences may still exist. Nevertheless, prior studies employing similar strategies have reported generally consistent findings (e.g., higher ISC during powerful speeches; Schmälzle et al., 2015), which to some extent supports the robustness of our findings. Looking forward, future research should incorporate a broader range of materials when resources permit. Such efforts can be facilitated by the growing availability of openly shared resources (Wang et al., 2025), which provide valuable datasets for large-scale investigations of audience engagement.

Interestingly, we observed dynamic changes in ISC within the language network across both speeches. An exploratory annotation of ISC peaks and troughs revealed that high ISC coincided with novel examples and engaging stories, whereas low ISC aligned with abstract or less captivating content (see Figure 6 and Table S6). These findings suggest that narrative features shape neural synchrony, which may also be influenced by factors such as lexical diversity, information density, and semantic or sentiment variations. Future studies could leverage natural language processing techniques and large language models to systematically test these possibilities. Beyond verbal content, our video stimuli also conveyed nonverbal cues (e.g., gestures, facial expressions, posture) and paraverbal cues (e.g., tone, prosody, rhythm). These multimodal features are integral to public speaking and likely contributed to the dynamic patterns of neural synchronization observed here. Considering these dimensions highlights the broader research potential of ISC for capturing how verbal, nonverbal, and paraverbal signals jointly shape audience engagement. Future work could further disentangle the contributions of these communicative channels, thereby linking linguistic, social, and affective aspects of speech more directly to underlying brain mechanisms.

Additionally, this study focused only on audience brain alignment and did not address the speaker’s cognitive or emotional processes or delivery strategies. Because public speaking is inherently live and interactive, future research should use methods such as wearable EEG or fNIRS in more naturalistic settings to capture speaker–audience dynamics and real-time interactions (Chang et al., 2024).

### Conclusion

Our study investigated the neurobiological correlates of successful public speaking by examining the spatial patterns and temporal dynamics of interbrain neural synchronization. We found that effective speeches elicited increased ISC across the brain, particularly those regions involved in language processing and social cognition. Converging evidence suggests that these engagement-related effects may be supported by underlying neurobiological processes involving synaptic communication and signaling. Our findings offer valuable insights into the neuroscience of effective communication and education.

## ACKNOWLEDGMENTS

The study protocol was approved by the Institutional Review Board of Zhejiang University. We thank the Information Technology Center of Zhejiang University for their support.

## FUNDING INFORMATION

Xiang-Zhen Kong, STI 2030-Major Projects, Award ID: 2021ZD0200409. Xiang-Zhen Kong, STI 2030-Major Projects, Award ID: 2025ZD0218800. Xiang-Zhen Kong, Fundamental Research Funds for the Central Universities, Award ID: 226-2025-00144. Xiang-Zhen Kong, National Natural Science Foundation of China (https://dx.doi.org/10.13039/501100001809), Award ID: 32171031 and 32571219. Yi Pu, National Natural Science Foundation of China (https://dx.doi.org/10.13039/501100001809), Award ID: 32400882.

## AUTHOR CONTRIBUTIONS

**Xuanxuan Zhang**: Conceptualization; Formal analysis; Project administration; Visualization; Writing – original draft; Writing – review & editing. **Bolong Wang**: Conceptualization; Formal analysis; Project administration; Visualization; Writing – original draft; Writing – review & editing. **Linmiao Zhang**: Conceptualization; Writing – review & editing. **Yi Pu**: Conceptualization; Funding acquisition; Resources; Writing – review & editing. **Xiang-Zhen Kong**: Conceptualization; Funding acquisition; Methodology; Resources; Writing – original draft; Writing – review & editing.

## DATA AND CODE AVAILABILITY STATEMENTS

The BIDS formatted data is available on OpenNeuro at the following link: https://openneuro.org/datasets/ds005920 (Wang et al., 2025). Related behavior data is available on GitHub: https://github.com/cognomicslab/naturalistic_stimuli. The code for ISC analysis is available on GitHub at the following link: https://github.com/cognomicslab/naturalistic_stimuli.

## Supplementary Material





## TECHNICAL TERMS

Intersubject correlation (ISC): Similarity of brain activity time courses across people viewing the same stimulus.
Generalized linear model (GLM): Statistical framework linking predictors to signals to estimate task-related effects in brain data.
GABAergic receptors: Proteins responding to GABA, the main inhibitory transmitter, reducing neurons’ likelihood to fire.
Naturalistic stimuli: Real-world materials, like movies or stories, mimicking everyday experience in experiments.
Language network: Brain regions supporting speech production and comprehension, typically in frontal and temporal lobes.
Gene set enrichment: Statistical test assessing whether predefined gene groups are overrepresented among significant genes.
GO terms: Standardized gene ontology (GO) labels for gene functions, biological processes, and cellular components.
Default mode network (DMN): Brain network engaged during rest, self-related thought, memory, and mind-wandering.
Functional connectivity: Statistical dependence between brain regions’ activity over time, indicating coordinated fluctuations.

## References

[bib1] Abrams, D. A., Ryali, S., Chen, T., Chordia, P., Khouzam, A., Levitin, D. J., & Menon, V. (2013). Inter-subject synchronization of brain responses during natural music listening. European Journal of Neuroscience, 37(9), 1458–1469. 10.1111/ejn.12173, 23578016 PMC4487043

[bib2] Achard, S., & Bullmore, E. (2007). Efficiency and cost of economical brain functional networks. PLOS Computational Biology, 3(2), Article e17. 10.1371/journal.pcbi.0030017, 17274684 PMC1794324

[bib3] Allen, M., Hunter, J. E., & Donohue, W. A. (1989). Meta-analysis of self-report data on the effectiveness of public speaking anxiety treatment techniques. Communication Education, 38(1), 54–76. 10.1080/03634528909378740

[bib4] Bolton, T. A. W., Freitas, L. G. A., Jochaut, D., Giraud, A.-L., & Van De Ville, D. (2020). Neural responses in autism during movie watching: Inter-individual response variability co-varies with symptomatology. NeuroImage, 216, Article 116571. 10.1016/j.neuroimage.2020.116571, 31987996

[bib5] Chang, C. H. C., Lazaridi, C., Yeshurun, Y., Norman, K. A., & Hasson, U. (2021). Relating the past with the present: Information integration and segregation during ongoing narrative processing. Journal of Cognitive Neuroscience, 33(6), 1106–1128. 10.1162/jocn_a_01707, 34428791 PMC9155984

[bib6] Chang, C. H. C., Nastase, S. A., Zadbood, A., & Hasson, U. (2024). How a speaker herds the audience: Multibrain neural convergence over time during naturalistic storytelling. Social Cognitive and Affective Neuroscience, 19(1), Article nsae059. 10.1093/scan/nsae059, 39223692 PMC11421471

[bib7] Chao-Gan, Y., & Yu-Feng, Z. (2010). DPARSF: A MATLAB toolbox for “pipeline” data analysis of resting-state fMRI. Frontiers in Systems Neuroscience, 4, Article 13. 10.3389/fnsys.2010.00013, 20577591 PMC2889691

[bib8] Chattopadhyaya, B., & Di Cristo, G. (2012). GABAergic circuit dysfunctions in neurodevelopmental disorders. Frontiers in Psychiatry, 3, Article 51. 10.3389/fpsyt.2012.00051, 22666213 PMC3364508

[bib9] Chen, L., Feng, G., Leong, C. W., Joe, J., Kitchen, C., & Lee, C. M. (2016). Designing an automated assessment of public speaking skills using multimodal cues. Journal of Learning Analytics, 3(2), 261–281. 10.18608/jla.2016.32.13

[bib10] Cohen, S. S., Henin, S., & Parra, L. C. (2017). Engaging narratives evoke similar neural activity and lead to similar time perception. Scientific Reports, 7(1), Article 4578. 10.1038/S41598-017-04402-4, 28676688 PMC5496904

[bib11] Czepiel, A., Fink, L. K., Fink, L. T., Wald-Fuhrmann, M., Tröndle, M., & Merrill, J. (2021). Synchrony in the periphery: Inter-subject correlation of physiological responses during live music concerts. Scientific Reports, 11(1), Article 22457. 10.1038/S41598-021-00492-3, 34789746 PMC8599424

[bib12] Davey, J., Thompson, H. E., Hallam, G., Karapanagiotidis, T., Murphy, C., De Caso, I., Krieger-Redwood, K., Bernhardt, B. C., Smallwood, J., & Jefferies, E. (2016). Exploring the role of the posterior middle temporal gyrus in semantic cognition: Integration of anterior temporal lobe with executive processes. NeuroImage, 137, 165–177. 10.1016/j.neuroimage.2016.05.051, 27236083 PMC4927261

[bib13] Dmochowski, J. P., Bezdek, M. A., Abelson, B. P., Johnson, J. S., Schumacher, E. H., & Parra, L. C. (2014). Audience preferences are predicted by temporal reliability of neural processing. Nature Communications, 5, Article 4567. 10.1038/ncomms5567, 25072833 PMC4124862

[bib14] Eden, E., Navon, R., Steinfeld, I., Lipson, D., & Yakhini, Z. (2009). *GOrilla*: A tool for discovery and visualization of enriched GO terms in ranked gene lists. BMC Bioinformatics, 10, Article 48. 10.1186/1471-2105-10-48, 19192299 PMC2644678

[bib15] Falk, E., & Scholz, C. (2018). Persuasion, influence, and value: Perspectives from communication and social neuroscience. Annual Review of Psychology, 69, 329–356. 10.1146/annurev-psych-122216-011821, 28961060 PMC12175252

[bib16] Farris, K., Houser, M., & Wotipka, C. (2013). Assessing student public speaking competence in the hybrid basic communication course. Basic Communication Course Annual, 25(1), Article 10. https://ecommons.udayton.edu/bcca/vol25/iss1/10

[bib17] Ferguson, B. R., & Gao, W.-J. (2018). PV interneurons: Critical regulators of E/I balance for prefrontal cortex-dependent behavior and psychiatric disorders. Frontiers in Neural Circuits, 12, Article 37. 10.3389/fncir.2018.00037, 29867371 PMC5964203

[bib18] Fotheringham, W. C. (1956). A technique for measuring speech effectiveness in public speaking classes. Speech Monographs, 23(1), 31–37. 10.1080/03637755609375162

[bib19] Fox, M. D., Snyder, A. Z., Vincent, J. L., Corbetta, M., Van Essen, D. C., & Raichle, M. E. (2005). The human brain is intrinsically organized into dynamic, anticorrelated functional networks. Proceedings of the National Academy of Sciences of the United States of America, 102(27), 9673–9678. 10.1073/pnas.0504136102, 15976020 PMC1157105

[bib20] Grall, C., Tamborini, R., Weber, R., & Schmälzle, R. (2021). Stories collectively engage listeners’ brains: Enhanced intersubject correlations during reception of personal narratives. Journal of Communication, 71(2), 332–355. 10.1093/joc/jqab004

[bib21] Haiman, F. S. (1949). An experimental study of the effects of ethos in public speaking. Speech Monographs, 16(2), 190–202. 10.1080/03637754909374974

[bib22] Hasson, U., Ghazanfar, A. A., Galantucci, B., Garrod, S., & Keysers, C. (2012). Brain-to-brain coupling: A mechanism for creating and sharing a social world. Trends in Cognitive Sciences, 16(2), 114–121. 10.1016/j.tics.2011.12.007, 22221820 PMC3269540

[bib23] Hasson, U., Landesman, O., Knappmeyer, B., Vallines, I., Rubin, N., & Heeger, D. J. (2008). Neurocinematics: The neuroscience of film. Projections, 2(1), 1–26. 10.3167/proj.2008.020102

[bib24] Hasson, U., Malach, R., & Heeger, D. J. (2010). Reliability of cortical activity during natural stimulation. Trends in Cognitive Sciences, 14(1), 40–48. 10.1016/j.tics.2009.10.011, 20004608 PMC2818432

[bib25] Hasson, U., Nir, Y., Levy, I., Fuhrmann, G., & Malach, R. (2004). Intersubject synchronization of cortical activity during natural vision. Science, 303(5664), 1634–1640. 10.1126/science.1089506, 15016991

[bib26] Hawrylycz, M. J., Lein, E. S., Guillozet-Bongaarts, A. L., Shen, E. H., Ng, L., Miller, J. A., van de Lagemaat, L. N., Smith, K. A., Ebbert, A., Riley, Z. L., Abajian, C., Beckmann, C. F., Bernard, A., Bertagnolli, D., Boe, A. F., Cartagena, P. M., Mallar Chakravarty, M., Chapin, M., Chong, J., … Jones, A. R. (2012). An anatomically comprehensive atlas of the adult human brain transcriptome. Nature, 489(7416), 391–399. 10.1038/nature11405, 22996553 PMC4243026

[bib27] Herbein, E., Golle, J., Tibus, M., Schiefer, J., Trautwein, U., & Zettler, I. (2018). Fostering elementary school children’s public speaking skills: A randomized controlled trial. Learning and Instruction, 55, 158–168. 10.1016/j.learninstruc.2017.10.008

[bib28] Hickok, G. (2014). The myth of mirror neurons: The real neuroscience of communication and cognition. W. W. Norton. 10.1177/1747021819876534 31476966

[bib29] Imhof, M. A., Schmälzle, R., Renner, B., & Schupp, H. T. (2017). How real-life health messages engage our brains: Shared processing of effective anti-alcohol videos. Social Cognitive and Affective Neuroscience, 12(7), 1188–1196. 10.1093/scan/nsx044, 28402568 PMC5490672

[bib30] Imhof, M. A., Schmälzle, R., Renner, B., & Schupp, H. T. (2020). Strong health messages increase audience brain coupling. NeuroImage, 216, Article 116527. 10.1016/j.neuroimage.2020.116527, 31954843

[bib31] Ki, J. J., Kelly, S. P., & Parra, L. C. (2016). Attention strongly modulates reliability of neural responses to naturalistic narrative stimuli. Journal of Neuroscience, 36(10), 3092–3101. 10.1523/JNEUROSCI.2942-15.2016, 26961961 PMC6601758

[bib32] Kleiner, M., Brainard, D., & Pelli, D. (2007). What’s new in Psychtoolbox-3? [Slide presentation]. Max Planck Institute for Biological Cybernetics.

[bib33] Lahnakoski, J. M., Glerean, E., Jääskeläinen, I. P., Hyönä, J., Hari, R., Sams, M., & Nummenmaa, L. (2014). Synchronous brain activity across individuals underlies shared psychological perspectives. NeuroImage, 100, 316–324. 10.1016/j.neuroimage.2014.06.022, 24936687 PMC4153812

[bib34] Li, Z., Hong, B., Nolte, G., Engel, A. K., & Zhang, D. (2024). Speaker–listener neural coupling correlates with semantic and acoustic features of naturalistic speech. Social Cognitive and Affective Neuroscience, 19(1), Article nsae051. 10.1093/scan/nsae051, 39012092 PMC11296674

[bib35] Lotter, L. D., Kohl, S. H., Gerloff, C., Bell, L., Niephaus, A., Kruppa, J. A., Dukart, J., Schulte-Rüther, M., Reindl, V., & Konrad, K. (2023). Revealing the neurobiology underlying interpersonal neural synchronization with multimodal data fusion. Neuroscience & Biobehavioral Reviews, 146, Article 105042. 10.1016/j.neubiorev.2023.105042, 36641012

[bib36] Lucas, S. (2004). The art of public speaking (8th ed.). McGraw-Hill.

[bib37] Markello, R. D., Arnatkeviciute, A., Poline, J.-B., Fulcher, B. D., Fornito, A., & Misic, B. (2021). Standardizing workflows in imaging transcriptomics with the abagen toolbox. eLife, 10, Article e72129. 10.7554/eLife.72129, 34783653 PMC8660024

[bib38] Markello, R. D., Hansen, J. Y., Liu, Z.-Q., Bazinet, V., Shafiei, G., Suárez, L. E., Blostein, N., Seidlitz, J., Baillet, S., Satterthwaite, T. D., Mallar Chakravarty, M., Raznahan, A., & Misic, B. (2022). neuromaps: Structural and functional interpretation of brain maps. Nature Methods, 19(11), 1472–1479. 10.1038/s41592-022-01625-w, 36203018 PMC9636018

[bib39] Martinez-Conde, S., Alexander, R. G., Blum, D., Britton, N., Lipska, B. K., Quirk, G. J., Swiss, J. I., Willems, R. M., & Macknik, S. L. (2019). The storytelling brain: How neuroscience stories help bridge the gap between research and society. Journal of Neuroscience, 39(42), 8285–8290. 10.1523/JNEUROSCI.1180-19.2019, 31619498 PMC6794920

[bib40] Mildner, V. (2008). The cognitive neuroscience of human communication. Psychology Press. 10.4324/9780203838105

[bib41] Nastase, S. A., Gazzola, V., Hasson, U., & Keysers, C. (2019). Measuring shared responses across subjects using intersubject correlation. Social Cognitive and Affective Neuroscience, 14(6), 667–685. 10.1093/scan/nsz037, 31099394 PMC6688448

[bib42] Nastase, S. A., Goldstein, A., & Hasson, U. (2020). Keep it real: Rethinking the primacy of experimental control in cognitive neuroscience. NeuroImage, 222, Article 117254. 10.1016/j.neuroimage.2020.117254, 32800992 PMC7789034

[bib43] Nguyen, M., Chang, A., Micciche, E., Meshulam, M., Nastase, S. A., & Hasson, U. (2022). Teacher–student neural coupling during teaching and learning. Social Cognitive and Affective Neuroscience, 17(4), 367–376. 10.1093/scan/nsab103, 34450637 PMC8972247

[bib44] Nguyen, M., Vanderwal, T., & Hasson, U. (2019). Shared understanding of narratives is correlated with shared neural responses. NeuroImage, 184, 161–170. 10.1016/j.neuroimage.2018.09.010, 30217543 PMC6287615

[bib45] Nichols, T. E., & Holmes, A. P. (2002). Nonparametric permutation tests for functional neuroimaging: A primer with examples. Human Brain Mapping, 15(1), 1–25. 10.1002/hbm.1058, 11747097 PMC6871862

[bib46] Noonan, K. A., Jefferies, E., Visser, M., & Lambon Ralph, M. A. (2013). Going beyond inferior prefrontal involvement in semantic control: Evidence for the additional contribution of dorsal angular gyrus and posterior middle temporal cortex. Journal of Cognitive Neuroscience, 25(11), 1824–1850. 10.1162/jocn_a_00442, 23859646

[bib47] Nummenmaa, L., Glerean, E., Viinikainen, M., Jääskeläinen, I. P., Hari, R., & Sams, M. (2012). Emotions promote social interaction by synchronizing brain activity across individuals. Proceedings of the National Academy of Sciences of the United States of America, 109(24), 9599–9604. 10.1073/pnas.1206095109, 22623534 PMC3386135

[bib48] Nummenmaa, L., Lahnakoski, J. M., & Glerean, E. (2018). Sharing the social world via intersubject neural synchronisation. Current Opinion in Psychology, 24, 7–14. 10.1016/j.copsyc.2018.02.021, 29550395

[bib49] Ohad, T., & Yeshurun, Y. (2023). Neural synchronization as a function of engagement with the narrative. NeuroImage, 276, Article 120215. 10.1016/j.neuroimage.2023.120215, 37269956

[bib50] Pérez, P., Madsen, J., Banellis, L., Türker, B., Raimondo, F., Perlbarg, V., Valente, M., Niérat, M.-C., Puybasset, L., Naccache, L., Similowski, T., Cruse, D., Parra, L. C., & Sitt, J. D. (2021). Conscious processing of narrative stimuli synchronizes heart rate between individuals. Cell Reports, 36(11), Article 109692. 10.1016/j.cekrep.2021.109692, 34525363

[bib51] Raichle, M. E. (2015). The brain’s default mode network. Annual Review of Neuroscience, 38, 433–447. 10.1146/annurev-neuro-071013-014030, 25938726

[bib52] Rodero, E. (2022). Effectiveness, attractiveness, and emotional response to voice pitch and hand gestures in public speaking. Frontiers in Communication, 7, Article 869084. 10.3389/fcomm.2022.869084

[bib53] Schaefer, A., Kong, R., Gordon, E. M., Laumann, T. O., Zuo, X.-N., Holmes, A. J., Eickhoff, S. B., & Yeo, B. T. T. (2018). Local–global parcellation of the human cerebral cortex from intrinsic functional connectivity MRI. Cerebral Cortex, 28(9), 3095–3114. 10.1093/cercor/bhx179, 28981612 PMC6095216

[bib54] Schmälzle, R. (2022). Theory and method for studying how media messages prompt shared brain responses along the sensation-to-cognition continuum. Communication Theory, 32(4), 450–460. 10.1093/ct/qtac009

[bib55] Schmälzle, R., & Grall, C. (2020). The coupled brains of captivated audiences: An investigation of the collective brain dynamics of an audience watching a suspenseful film. Journal of Media Psychology: Theories, Methods, and Applications, 32(4), 187–199. 10.1027/1864-1105/A000271

[bib56] Schmälzle, R., Häcker, F. E. K., Honey, C. J., & Hasson, U. (2015). Engaged listeners: Shared neural processing of powerful political speeches. Social Cognitive and Affective Neuroscience, 10(8), 1137–1143. 10.1093/scan/nsu168, 25653012 PMC4526488

[bib57] Schmälzle, R., Lim, S., Du, Y., & Bente, G. (2025). The art of audience engagement: LLM-based thin-slicing of scientific talks. Frontiers in Communicaton, 10, Article 1610404. 10.3389/fcomm.2025.1610404

[bib58] Schmälzle, R., Liu, H., Delle, F. A., Lewin, K. M., Jahn, N. T., Zhang, Y., Yoon, H., & Long, J. (2024). Moment-by-moment tracking of audience brain responses to an engaging public speech: Replicating the reverse-message engineering approach. Communication Monographs, 91(1), 31–55. 10.1080/03637751.2023.2240398

[bib59] Schmälzle, R., Wilcox, S., & Jahn, N. T. (2022). Identifying moments of peak audience engagement from brain responses during story listening. Communication Monographs, 89(4), 515–538. 10.1080/03637751.2022.2032229

[bib60] Schmidt-Wilcke, T., Fuchs, E., Funke, K., Vlachos, A., Müller-Dahlhaus, F., Puts, N. A. J., Harris, R. E., & Edden, R. A. E. (2018). GABA—From inhibition to cognition: Emerging concepts. Neuroscientist, 24(5), 501–515. 10.1177/1073858417734530, 29283020

[bib61] Schreiber, L. M., Paul, G. D., & Shibley, L. R. (2012). The development and test of the Public Speaking Competence Rubric. Communication Education, 61(3), 205–233. 10.1080/03634523.2012.670709

[bib62] Seghier, M. L. (2013). The angular gyrus: Multiple functions and multiple subdivisions. Neuroscientist, 19(1), 43–61. 10.1177/1073858412440596, 22547530 PMC4107834

[bib63] Song, H., Finn, E. S., & Rosenberg, M. D. (2021). Neural signatures of attentional engagement during narratives and its consequences for event memory. Proceedings of the National Academy of Sciences of the United States of America, 118(33), Article e2021905118. 10.1073/pnas.2021905118, 34385312 PMC8379980

[bib64] Sonkusare, S., Breakspear, M., & Guo, C. (2019). Naturalistic stimuli in neuroscience: Critically acclaimed. Trends in Cognitive Sciences, 23(8), 699–714. 10.1016/j.tics.2019.05.004, 31257145

[bib65] Stolk, A., Verhagen, L., & Toni, I. (2016). Conceptual alignment: How brains achieve mutual understanding. Trends in Cognitive Sciences, 20(3), 180–191. 10.1016/j.tics.2015.11.007, 26792458

[bib66] Thomas Yeo, B. T., Krienen, F. M., Sepulcre, J., Sabuncu, M. R., Lashkari, D., Hollinshead, M., Roffman, J. L., Smoller, J. W., Zöllei, L., Polimeni, J. R., Fisch, B., Liu, H., & Buckner, R. L. (2011). The organization of the human cerebral cortex estimated by intrinsic functional connectivity. Journal of Neurophysiology, 106(3), 1125–1165. 10.1152/jn.00338.2011, 21653723 PMC3174820

[bib67] Todorov, A. (2017). Face value: The irresistible influence of first impressions. Princeton University Press. 10.1515/9781400885725

[bib68] van Baar, J. M., Halpern, D. J., & FeldmanHall, O. (2021). Intolerance of uncertainty modulates brain-to-brain synchrony during politically polarized perception. Proceedings of the National Academy of Sciences of the United States of America, 118(20), Article e2022491118. 10.1073/pnas.2022491118, 33986114 PMC8157931

[bib69] Wang, B., Zhang, X., Zhang, L., & Kong, X.-Z. (2025). A naturalistic fMRI dataset in response to public speaking. Scientific Data, 12(1), Article 659. 10.1038/S41597-025-05017-5, 40253420 PMC12009387

[bib70] Willems, R. M., Nastase, S. A., & Milivojevic, B. (2020). Narratives for neuroscience. Trends in Neuroscience, 43(5), 271–273. 10.1016/j.tins.2020.03.003, 32353331

[bib71] Wilson, S. M., Molnar-Szakacs, I., & Iacoboni, M. (2008). Beyond superior temporal cortex: Intersubject correlations in narrative speech comprehension. Cerebral Cortex, 18(1), 230–242. 10.1093/cercor/bhm049, 17504783

[bib72] Yarkoni, T., Poldrack, R. A., Nichols, T. E., Van Essen, D. C., & Wager, T. D. (2011). Large-scale automated synthesis of human functional neuroimaging data. Nature Methods, 8(8), 665–670. 10.1038/nmeth.1635, 21706013 PMC3146590

[bib73] Yeshurun, Y., Swanson, S., Simony, E., Chen, J., Lazaridi, C., Honey, C. J., & Hasson, U. (2017). Same story, different story: The neural representation of interpretive frameworks. Psychological Science, 28(3), 307–319. 10.1177/0956797616682029, 28099068 PMC5348256

[bib74] Zacks, J. M., & Magliano, J. P. (2013). Film, narrative, and cognitive neuroscience. In D. P. Melcher & F. Bacci (Eds.), Art and the senses (pp. 435–454). Oxford University Press.

[bib75] Zeng, H., Wang, X., Wang, Y., Wu, A., Pong, T.-C., & Qu, H. (2023). *GestureLens*: Visual analysis of gestures in presentation videos. IEEE Transactions on Visualization and Computer Graphics, 29(8), 3685–3697. 10.1109/TVCG.2022.3169175, 35446768

