# Supplementary material for: Successful Public Speaking Enhances Neural Alignment in Audience Language Networks
Source: Neurobiol Lang (Camb). 2026 Feb 20;7:NOL.a.218. doi: 10.1162/NOL.a.218 (PMC12978675; doi:10.1162/NOL.a.218)
Supplement: Supplementary file 1 [file nol-07-218-s001.pdf]

Supplementary Information

Supplementary Tables 1-6

Table S1. 30 candidate speech videos and detailed information.

Number	Title	Time	Topic	Duration	Link
1	What are we living for	2022.07.16	Literature	28'54"	https://www.yixi.tv/#/speech/detail?id=1103
2	How to approach love in the golden age of singleness	2017.06.10	Love	31'58"	https://www.yixi.tv/#/speech/detail?id=338
3	A long-term visual investigator	2018.08.26	Photography	27'21"	https://www.yixi.tv/#/speech/detail?id=685
4	The prism of solitude	2018.08.26	Literature	31'18"	https://www.yixi.tv/#/speech/detail?id=688
5	The truth that comes with time	2019.10.27	Literature	30'02"	https://www.yixi.tv/#/speech/detail?id=832
6	This story is entirely non-fiction	2016.12.22	Life	35'06"	https://www.yixi.tv/#/speech/detail?id=153
7	How can we draw a hedgehog?	2019.12.28	Nature	35'14"	https://www.yixi.tv/#/speech/detail?id=868
8	What a more complete healthcare system looks like	2023.01.08	Health	32'22"	https://www.yixi.tv/#/speech/detail?id=1149

9	What is science	2017.<br>03.19	Scienc<br>e	29'2<br>0"	https://www.yixi.tv/#/<br>speech/detail?id=144
10	What's wrong with our design	2018.<br>04.15	Design	24'1<br>3"	https://www.yixi.tv/#/<br>speech/detail?id=646
11	Thank you for giving me the chance to take the stage	2014.<br>09.07	Life	34'4<br>6"	https://www.yixi.tv/#/<br>speech/detail?id=114
12	How Bill Gates spends his money	2018.<br>07.15	Societ<br>y	30'0<br>7"	https://www.yixi.tv/#/<br>speech/detail?id=780
13	Cordelia's fate	2017.<br>10.22	Literat<br>ure	27'1<br>9"	https://www.yixi.tv/#/<br>speech/detail?id=586
14	What reading really is	2016.<br>11.27	Cultur<br>e	22'1<br>8"	https://www.yixi.tv/#/<br>speech/detail?id=298
15	The psychology behind anti-human-centered design	2018.<br>12.15	Psych<br>ology	26'5<br>9"	https://www.yixi.tv/#/<br>speech/detail?id=741
16	Material heroes	2019.<br>12.28	Design	26'3<br>2"	https://www.yixi.tv/#/<br>speech/detail?id=864
17	The urban tracker	2018.<br>03.11	City	36'4<br>7"	https://www.yixi.tv/#/<br>speech/detail?id=620
18	The importance of salt reduction	2019.<br>10.27	Health	26'0<br>0"	https://www.yixi.tv/#/<br>speech/detail?id=841
19	The prince and the ruins	2022.<br>09.25	Literat<br>ure	26'0<br>2"	https://www.yixi.tv/#/<br>speech/detail?id=1127

20	Urban breathing	2016.10.30	Architecture	25'55"	https://www.yixi.tv/#/speech/detail?id=120
21	Moss never disappears	2017.08.19	Literature	24'39"	https://www.yixi.tv/#/speech/detail?id=579
22	Clothes, emotions, and women	2017.08.19	Design	20'40"	https://www.yixi.tv/#/speech/detail?id=560
23	The self-cultivation of a young wild spirit	2015.09.20	Nature	31'51"	https://www.yixi.tv/#/speech/detail?id=185
24	Grateful to have met you	2017.04.08	Photography	24'04"	https://www.yixi.tv/#/speech/detail?id=139
25	Epiphyllum bloom	2016.12.22	Film	31'42"	https://www.yixi.tv/#/speech/detail?id=40
26	Mai Po	2017.07.15	Environment	21'29"	https://www.yixi.tv/#/speech/detail?id=565
27	The comic sage	2020.08.23	Imagination	31'13"	https://www.yixi.tv/#/speech/detail?id=906
28	Choices at Lashi Lake	2017.04.08	Anthropology	31'31"	https://www.yixi.tv/#/speech/detail?id=122
29	Gazing at oneself with a curious mind	2013.10.20	Anthropology	20'47"	https://www.yixi.tv/#/speech/detail?id=236
30	Bigger	2016.10.30	Design	24'28"	https://www.yixi.tv/#/speech/detail?id=303

Table S2. Candidate speech videos.

Number	Title	Time	Topic	Duration	Link
**1**	What's wrong with our design	2018.04.15	Design	24'13"	https://www.yixi.tv/#/speech/detail?id=646
**2**	Bigger	2016.10.30	Design	24'28"	https://www.yixi.tv/#/speech/detail?id=303
**3**	The truth that comes with time	2019.10.27	Literature	30'02"	https://www.yixi.tv/#/speech/detail?id=832
**4**	The prince and the ruins	2022.09.25	Literature	26'02"	https://www.yixi.tv/#/speech/detail?id=1127

Table S3. Speech rating questionnaire.

Number	Question
**Q1**	Your overall impression of this speech is very positive.
**Q2**	You have a high level of comprehension of this speech.
**Q3**	You agree with the viewpoints of this speech.
**Q4**	You have a high level of emotional resonance with this speech.

**Q5**	The speaker's appearance is excellent.
**Q6**	The speaker's facial expression is appropriate.
**Q7**	The speaker's body language is appropriate.
**Q8**	The speaker's intonation is appropriate.
**Q9**	The speaker's pronunciation is excellent.
**Q10**	The speaker's speaking rate is appropriate.
**Q11**	The speech's content is persuasive.
**Q12**	The speech's content is clear.
**Q13**	The speech's content is well-organized.
**Q14**	The speech's content is insightful.
**Q15**	The speech's content is novel in its viewpoints.
**Q16**	The speech's content is vivid and imaginative.
**Q17**	The speech's content is engaging.
**Q18**	The speech's colloquial style is appropriate.

Note: The questionnaire consisted of 18 questions, each rated on a five-point Likert scale. Participants were instructed to evaluate each question based on their agreement, using the following options: "Strongly Disagree," "Disagree," "Neutral," "Agree," and "Strongly Agree," corresponding to scores of 1, 2, 3, 4, and 5, respectively.

Table S4. Comprehension questions for the HSS and the LSS.

Speech	Question	Options

**HSS**	The speaker thinks that art is ( ).	A. For me (correct answer)<br>B. For you
**LSS**	The speaker's attitude towards the emergence of many exaggerated designs in the current fashion industry is ( ).	A. Positive<br>B. Negative (correct answer)

Table S5. Neurosynth decoding results: functions showing top associations with corresponding ISC maps.

**HSS**		**LSS**		**t-test statistic (HSS>LSS)**		**t-test statistic (HSS<LSS)**	
visual	0.525	visual	0.640	language	0.255	fusiform	-0.191
occipital	0.437	occipital	0.583	comprehension	0.241	fusiform gyrus	-0.174
motion	0.372	fusiform	0.474	sentence	0.247	face	-0.165
v1	0.361	objects	0.461	theory mind	0.238	fusiform face	-0.157
extrastriate	0.343	occipito	0.419	sentences	0.228	faces	-0.154
mt	0.315	fusiform gyrus	0.414	middle temporal	0.227	face ffa	-0.147
visual cortex	0.313	object	0.385	mind tom	0.220	objects	-0.143
v5	0.309	face	0.374	temporal	0.220	ffa	-0.141
objects	0.305	occipito temporal	0.372	temporal sulcus	0.220	somatosensory	-0.140
object	0.296	extrastriate	0.353	mind	0.218	occipital	-0.138
perception	0.284	occipitotemporal	0.344	linguistic	0.218	primary motor	-0.128
lateral occipital	0.283	ventral visual	0.327	inferior frontal	0.218	primary	-0.127

occipitotemporal	0.281	lateral occipital	0.319	semantic	0.218	occipito	-0.123
occipito	0.279	fusiform face	0.318	tom	0.212	sensorimotor	-0.116
visual motion	0.278	faces	0.309	mental states	0.210	fusiform gyri	-0.115
occipito temporal	0.271	face ffa	0.307	inferior	0.207	motor cortex	-0.114
vision	0.265	ffa	0.299	posterior superior	0.206	ipsilateral	-0.112
occipital cortex	0.261	v1	0.287	frontal	0.189	face recognition	-0.112
fusiform	0.257	occipital cortex	0.282	mentalizing	0.189	visual	-0.111
temporal sulcus	0.257	occipitotemporal cortex	0.273	social	0.188	motor	-0.109
sulcus	0.250	visual cortex	0.261	phonological	0.186	primary somatosensory	-0.109
early visual	0.249	fusiform gyri	0.250	syntactic	0.185	ventral visual	-0.107
reading	0.243	visual stream	0.247	superior temporal	0.181	hand	-0.107
temporal	0.239	recognition	0.227	psts	0.176	contralateral	-0.106
occipitotemporal cortex	0.224	viewing	0.226	frontal gyrus	0.175	somatosensory cortex	-0.105

Table S6. Chinese-version and English-version of speech transcripts corresponding to ISC peaks and troughs for HSS and LSS.

HSS Peak	time-window	Chinese-version	English-version

**1**	**60-95s**	所以走进厕所，我一开门进去，第一个让我这个做设计做了这么久，有点怀疑自己的就是它那个男女厕所的标志。它画得非常简单，一条线，一个这样，一个这样。旁边没有人，两个紫颜色并且非常小，又都是大理石的。米色的。我在那看半天，我是应该进这个还是那个呢。然后我就叫了一声，先中文，有没有人？里面没有回答。Anybody there? 没有声音。我推门进去，很大。那个卫生间大概有这个台四个这么大。很大的一个卫生间，都是准备博物馆级的。一走进去以后呢，没灯。我就呆住了。然后我发现我一走，哎，灯亮了。然后我再走第二层。小便的那个空间大概有，我估计有十个小便器。	**So when I entered the restroom, the very first thing that made me, someone who's been in design for so long, doubt myself was the men's and women's restroom signs. They were drawn very simply: one line like this, one like that. There was no one around. The signs were small, purple, set against the marble walls which were beige. I stood there for a while, wondering which door I should enter. Then I called out, first in Chinese, "Is anyone there?" No answer inside. "Anybody there?" Still no sound. I pushed a door open; it was huge inside. The restroom was about four times the size of this stage. A very large restroom, furnished like a museum-grade space. But when I walked in, there were no lights on. I froze. Then I realized as I moved, the lights came on. Then I went to the next area. The urinal section had, I estimate, about ten urinals.**

2	335-360s	中国的设计，这个发展是我真正见证的，因为我是应该属于中国最早的去推动现代设计的那一代人。我记得我第一次来广东的时候，在广州美术学院。那时候还没有这个地方呢，华侨城也还没有呢，这个剧院也没有呢。当时深圳的边缘就是在现在岗厦那个地方。	I have personally witnessed the development of design in China, as I belong to that first generation who actively promoted modern design here. I remember when I first came to Guangdong, it was to the Guangzhou Academy of Fine Arts. At that time, this place didn't exist yet; OCT hadn't been built, and this theater wasn't here either. Back then, the edge of Shenzhen was around where Gangxia is now.
3	835-880s	如果你把这个海报搞成，哇，满天都是符号，几层不同的颜色。字体是反的，哎呀，肯定得大奖。这个是现在很常见的。工业设计展，基本上是不能用的东西。工业设计得大奖的东西拿出来，一根麻绳绑了一个木片。告诉你这个是生态的，环保的。最后你看一下，不错，给它打一个甲等奖，完了以后这个东西肯定是落选的。因为市场上卖的，我们现在	But if you make the poster, wow, covered in symbols, multiple layers of colors, reversed typeface – ah, it's guaranteed a top prize. This is very common now. Industrial design exhibitions are full of things that are basically unusable. The things that win major industrial design awards? Take one: merely a piece of wood tied with a hemp rope. They tell you it's ecological, environmentally friendly. In the end, you look at it, say "not bad,"

		看的手机东西绝对不走这一路。这就变成了一个问题，就是我们设计教育在做什么呢？	**award it the top prize, and then that thing will undoubtedly be rejected in the real world. The phones currently on the market, the ones we all use, absolutely don’t follow this path. This raises a question: What exactly is our design education doing?**
**4**	**1045-1075s**	哎，他们说小了好像不划算。这个水嘛，肯定要多是吧。 我想我觉得最好看的状态。 如果我在这里喝水，是拿一个透明的玻璃杯在这里喝，我觉得比较雅。我不知道各位有没有这个感觉。可能有人说要个功夫茶杯比较雅。我呢觉得是一个玻璃杯，这对我是最好的一个感觉。那么这个矿泉水的瓶子， 第一个做小一点， 第二个能不能把这个瓶子做成两节。	**But they argued smaller sizes aren’t cost-effective."Water volume must be substantial, right?" I envisioned the most elegant solution: Me drinking water from a transparent glass—that feels refined. I wonder if you share this feeling. Some might prefer a tiny tea cup. For me, a simple glass feels ideal. So for bottled water... First, make it smaller. Second, could the bottle have two sections?**

**5**	**1385-1425s**	**但不管怎么说，我们这个设计走了这么大一圈走到现在， 其实应该，我们说走过了那个虚华的阶段，我们现在应该回到零，考虑设计是为什么。 设计是真正是要教育一代年轻人， 做企业未来，开发研发创意的后备军。他有 flexibility，有弹性、有潜力、有想象力。 而他有一种为人民服务的 humble 的 feeling。这个是我讲的核心，谢谢各位。**	**Regardless, after all these years of design evolution......having passed through superficial extravagance, we should reset to zero——and reconsider design's purpose.Design must truly educate a new generation—cultivating a creative reserve force for enterprise. They need flexibility, resilience, potential, and imagination. And they must possess a humble attitude toward serving the people. This is my core message. Thank you all.**
**HSS Trough**	**time-window**	**Chinese-version**	**English-version**
**1**	**205-265s**	**哎呀，太愉快了。那就走进去，走进一发觉也有。因为我动手它就会开灯。这个有些时候你动手是不想开灯。大家知道吧。你不想它开，它也开。那就不用说了。晚上非常安静地洗了澡，脱了衣服睡觉。然后它就开始，真是，整个就暗下来了，就睡得很舒服**	**Oh, how delightful! So you walk in, and you do find... well, there are switches. Because when I moved my hand, the lights came on. Sometimes you move your hand *not* wanting the lights on. You know? You don't want them on, but they come on anyway. Needless to say. That night I took a quiet shower, got undressed,**

		了。半夜要起床，一起床整个房的灯都亮了，就是你自己在床上突然间觉得。因为大家知道，半夜起来你是想朦朦胧胧的有点灯，上个厕所再睡觉，就不要醒。结果它全亮了。你知道我这个年龄，全亮了我大概得一个钟头才睡着。然后再睡到早上六点钟的时候，有一点想上厕所，那个时候就跟自己说，不要上。	**and went to bed. Then the room really did gradually dim completely, and I slept very comfortably.**<br>**In the middle of the night, I needed to get up. As soon as I got up, all the lights in the room came on brightly. It was startling just lying there in bed. Because, you know, when you get up at night, you want just a little dim light to go to the bathroom and then back to sleep without fully waking up. Instead, everything was brightly lit. At my age, with all the lights on, it would take me about an hour to fall back asleep.**<br>**So when I woke up again around 6 AM needing to use the bathroom, I told myself: "Don't go."**
**2**	**660-685s**	中国现在有七百所大学有艺术与设计专业。大家都开，并且都开博士硕士课程。招的学生，每个学院一年招生就招几千人，多的上万人，很吓人呢。中国美术	**China now has seven hundred universities with Art and Design programs. They all offer them, and they all offer doctoral and master's programs. The number of students enrolled? Each**

		学院每年的考生是 16 万人。16 万人去学校考素描，每个人喝两瓶矿泉水就是 32 万瓶矿泉水。加上他的爹妈各喝两瓶，每一天消耗就是一百万矿泉水。	school enrolls thousands per year, some even tens of thousands. It's terrifying. China Academy of Art gets 160,000 applicants every year. 160,000 people go to the school to take the sketching exam. If each drinks two bottles of mineral water, that's 320,000 bottles. Add their parents each drinking two bottles, and that's a million bottles consumed in a single day.
3	735-765s	八个美术学院，八个艺术学院。每个学校都是 1 比 11，比 8，甚至 1 比 4 的这样的比例。中央美院长期 1 比 7，是这么一个比例。在这种比例里面，当然这个汤就煮得非常浓。大家知道我们老师就好像一把盐，学校就好比一锅汤。这锅汤放一把盐，这个汤是鲜味的，是吧？现在我们这个汤，开始变成一个水缸，还是放一把盐。现在最恐怖是，按照现在 250 万艺术设计的学生，我们	Eight fine arts academies and eight arts institutes. Each school had ratios like 1:11, 1:8, even 1:4. CAFA (Central Academy of Fine Arts) long maintained a 1:7 ratio. With ratios like that, naturally, the "soup" was very rich. You see, teachers are like a handful of salt, and the school is like a pot of soup. Put that handful of salt into the pot, and the soup is flavorful, right? Now our "soup" has become a water vat, yet we still only put in that same handful of salt. Now the most

		现在不是一个水缸的汤，我们是一个游泳池，放一把盐。	terrifying part is, with 2.5 million art and design students today, we're not talking a vat anymore; we're talking a swimming pool, with just one handful of salt.
4	1205-1240s	我们讲今天的设计出了什么事，就是整个的一个基础。我们现在很多人端久了，老觉得自己是个大师。我经常开会就见到这么一群大师。谎话很可怕，因为讲多了自己都信以为真。我现在看设计界全是信以为真的人。这些人出来评判，来教导学生，绝对是贻害。	What's wrong with design today stems from its foundation. Many posture so long they start believing they're masters. I often see such "masters" at conferences. Lies are dangerous—repeat them enough and you start believing them. The design world now seems full of true believers. When they judge and teach students, it's disastrous.
LSS Peak	time-window	Chinese-version	English-version
1	0-75s	大家好，我是张达，欢迎大家来到一席。我今天讲的这个题目是“更大”，为什么要讲这个题目呢？是因为最近一两年在时装流行领域里头有一些设计师做出了非常超大或者超长的衣服，我在这里试图来讨论一下在这	Hello everyone, I’m Zhang Da. Welcome to Yi Xi. Today’s topic is “Bigger.” Why am I talking about this? Over the past year or two in the world of fashion, some designers have produced garments that are extremely oversized or extraordinarily elongated, and here I aim

		个行为背后的一些原因。我先解释一下，讲四个小现象。第一个是我们经常可以在野外生存手册里看到，如果人遇到大型掠食动物的时候应该怎么做。当然这种手册通常会告诉你，克制住自己想逃跑的那种愿望，然后站着不动，尽可能把自己的衣服或者是帽子或者睡袋张开，使自己的轮廓变得非常大，弄出尽可能大的声响来吓退这个动物。	**to explore some of the reasons behind that behavior. First, let me explain by describing four small phenomena. Such guides typically advise you to suppress any urge to flee, stand your ground, spread out whatever you have—jacket, hat, or sleeping bag—to make your silhouette as large as possible, and then make the loudest noise you can to scare the animal away.**
**2**	**120-150s**	第三种情况，是动物在求偶时的反应。这是松鸡，在求偶的时候，它会把胸前的囊鼓胀起来，把尾巴的羽毛立起来，整个身体的轮廓就会加大，同时再加上鸣叫，会吸引异性的关注和注意，从而获得更多的求偶机会。	**The third phenomenon is the mating display.Here is the capercaillie: during courtship, it inflates the sac on its chest, fans up its tail feathers, enlarging its entire silhouette, and adds loud calls to attract the attention of potential mates, thereby securing more mating opportunities.**

3	1080-1115s	这是建筑方面的例子：尼泊尔传统木砖结构的建筑窗户很小；柯布西耶的萨伏伊别墅因钢梁支撑可开大跨度窗；赫尔佐格在纽约的高层公寓则可实现落地窗。它们的递进说明技术让尺度超越人手极限，推动人类在设计中不断挑战和突破。	Here is an architectural example: traditional Nepalese wood-and-brick buildings have small windows; in Le Corbusier's Villa Savoye, steel supports allow large-span windows; Herzog & de Meuron's high-rise in New York features floor-to-ceiling glass.Their progression shows how technology surpasses human hand limitations, driving continual challenges and breakthroughs in design.
LSS Trough	time-window	Chinese-version	English-version
1	490-535s	因此，传播完全在屏幕上进行；过去几十年的软件开发，让图像还原度和逼真度大幅提升，观众更愿意欣赏这些赏心悦目的画面，而非枯燥的文字。在这种传播和阅读方式下，什么内容最占优势？显然是图像，因为我们的大脑解析一幅图像比解析文字要轻松许多，这促使设计师更愿意将设计图像化。另一个外部因	Consequently, communication happens entirely on screens; decades of software development have vastly improved image fidelity and realism, making audiences prefer these visually pleasing images over dull text.Within this mode of communication and reading, what stands out most? Clearly, images take precedence, as our brains process an image much more easily than text, which

		素是所谓的注意力经济和粉丝经济。这里有两张图片，来自不同年代：一张是六十年代的街头围观，另一张是网络截图，显示几千人同时点赞。这两种场景的共同点是“围观”——一种线下的真实围观场面，另一种数字时代的在线围观。而因为数字技术和网络技术的发展，一个人或一件事可以被更大范围看到，这形成了一种“被关注”的资源，并转化为商业价值。	encourages designers to make their designs more visual. Another external factor is the so called attention economy and fan economy. Here are two images from different eras: one shows a street crowd in the 1960s, the other a screenshot from the internet where thousands have simultaneously liked a post.What they share is “spectatorship”—one being a real world crowd watching someone wearing an unusual outfit, the other a digital crowd of thousands liking someone’s online post.As digital and network technologies advance, a person or event can be seen by a much larger audience, creating the resource of “attention,” which translates into commercial value.
2	645-690s	我认为，这二十年来传播方式的改变，是设计师有意无意中选择这种方式凸显产品的主要原因之一。另一个内在原因是人类体	I believe that changes in communication over the past two decades are one of the main reasons designers, intentionally or not, choose this method to highlight their

		内潜藏的最原始动机。接下来要讨论的是“正常”与“反常”的话题，正是针对超大尺寸这一看似反常的现象进行探讨。我们首先来看一下“通常”是什么情况，因为我们讨论的是超大尺寸的服装。	**products.Another internal reason is the primal drive hidden within the human body.Next, I want to discuss the topic of “normal” versus “abnormal,” specifically examining the seemingly abnormal phenomenon of oversized proportions.First, let us quickly outline what is considered “usual,” since we are talking about oversized garments.**
**3**	**1015-1055s**	机器制造的布料宽度可以达到 1.5 米甚至更宽，为服装裁剪提供了更大的可能性，减少了拼接的需要。因此，我认为这是技术进步与人类渴望挑战极限的愿望相结合，为我们的生活创造了更多便利。	**Fabric produced by machines can reach 1.5 meters in width, or even wider, which offers greater possibilities when cutting clothes and reduces the need for piecing seams. Therefore, I believe this is an example of how technological progress, combined with humanity's desire to challenge its limits, creates more convenience in our lives.**

Supplementary Video 1. ISC dynamics across the brain along sliding time-windows.
